# A human monoclonal antibody blocks malaria transmission and defines a highly conserved neutralizing epitope on gametes

**DOI:** 10.1038/s41467-021-21955-1

**Published:** 2021-03-19

**Authors:** Camila H. Coelho, Wai Kwan Tang, Martin Burkhardt, Jacob D. Galson, Olga Muratova, Nichole D. Salinas, Thiago Luiz Alves e Silva, Karine Reiter, Nicholas J. MacDonald, Vu Nguyen, Raul Herrera, Richard Shimp, David L. Narum, Miranda Byrne-Steele, Wenjing Pan, Xiaohong Hou, Brittany Brown, Mary Eisenhower, Jian Han, Bethany J. Jenkins, Justin Y. A. Doritchamou, Margery G. Smelkinson, Joel Vega-Rodríguez, Johannes Trück, Justin J. Taylor, Issaka Sagara, Sara A. Healy, Jonathan P. Renn, Niraj H. Tolia, Patrick E. Duffy

**Affiliations:** 1 Pathogenesis and Immunity Section, Laboratory of Malaria Immunology and Vaccinology, National Institute of Allergy and Infectious Diseases, National Institutes of Health, Bethesda, MD USA; 2 Host-Pathogen Interactions and Structural Vaccinology Section, Laboratory of Malaria Immunology and Vaccinology, National Institute of Allergy and Infectious Diseases, National Institutes of Health, Bethesda, MD USA; 3 Vaccine Development Unit, Laboratory of Malaria Immunology and Vaccinology, National Institute of Allergy and Infectious Diseases, National Institutes of Health, Bethesda, MD USA; 4 Division of Immunology and Children’s Research Center, University Children’s Hospital Zurich, University of Zurich (UZH), Zurich, Switzerland; 5 Alchemab Therapeutics Ltd, 55-56 Russell Square, London, UK; 6 Laboratory of Malaria and Vector Research, National Institute of Allergy and Infectious Diseases, National Institutes of Health, Rockville, MD USA; 7 iRepertoire Inc., Huntsville, AL USA; 8 Biological Imaging Section, National Institute of Allergy and Infectious Diseases, National Institutes of Health, Bethesda, MD USA; 9 Fred Hutchinson Cancer Research Center, Seattle, WA USA; 10 Malaria Research and Training Center, University of Sciences, Techniques, and Technology, Bamako, Mali

**Keywords:** Protein vaccines, Parasite host response

## Abstract

Malaria elimination requires tools that interrupt parasite transmission. Here, we characterize B cell receptor responses among Malian adults vaccinated against the first domain of the cysteine-rich 230 kDa gamete surface protein Pfs230, a key protein in sexual stage development of *P. falciparum* parasites. Among nine Pfs230 human monoclonal antibodies (mAbs) that we generated, one potently blocks transmission to mosquitoes in a complement-dependent manner and reacts to the gamete surface; the other eight show only low or no blocking activity. The structure of the transmission-blocking mAb in complex with vaccine antigen reveals a large discontinuous conformational epitope, specific to domain 1 of Pfs230 and comprising six structural elements in the protein. The epitope is conserved, suggesting the transmission-blocking mAb is broadly functional. This study provides a rational basis to improve malaria vaccines and develop therapeutic antibodies for malaria elimination.

## Abstract (web summary)

Vaccines that interrupt malaria transmission will be important tools for malaria elimination. Here the authors identify a human monoclonal antibody from Pfs230 vaccinated individuals that blocks transmission of *Plasmodium falciparum* to mosquitoes in a complement-dependent manner and reacts with gamete surface.

## Introduction

Malaria eradication is a global priority and will require innovative strategies that, in addition to preventing or controlling human infection, might block parasite transmission through mosquitoes^1–3^. Malaria transmission-blocking vaccines (TBVs) are novel tools designed to target the stages of *Plasmodium falciparum* that initiate infection in the mosquito vector. TBV could be employed to reduce parasite transmission and pursue malaria elimination^4^.

Pfs230 is a leading TBV candidate present on the surface of *P. falciparum* gametocytes and gametes that mediates binding of exflagellating microgametes to red blood cells. This 230 kDa gamete surface protein is comprised of fourteen 6-cysteine (6-Cys) domains^1–3^, and parasites lacking this protein cannot bind to red blood cells or further develop into oocysts^1^. The functional activity of TBVs is strongly associated with antibodies^5^, and murine monoclonal antibodies against recombinant domain 1 of Pfs230 were determined to be functional and to bind gametes^6^, but no information on the identity or properties of human antibodies elicited in response to Pfs230 domain 1 (Pfs230D1) has been reported.

Sequences of matched heavy and light chain variable regions from single human B cells have been used to identify antibodies generated in response to infection or vaccination and to inform vaccinology^7–9^. In this study, we apply this approach to examine human antibodies elicited in response to Pfs230 TBV, specifically Pfs230D1 conjugated with the carrier ExoProtein A (EPA) and formulated in Alhydrogel^®^ (Pfs230D1-EPA/Alhydrogel^®^). Our results reveal that the vaccine can generate a high affinity transmission-blocking antibody that binds a conserved epitope on Pfs230D1. These findings support further development of TBV strategies to induce potent antibody responses against mosquito sexual stage parasites.

## Results and discussion

### Among nine Pfs230D1 human mAbs, only LMIV230-01 potently blocks parasite transmission to mosquitoes

We collected Pfs230D1-specific single memory B cells (Supplementary Figs. 1, 2, 3a) from eight Malian adults immunized with four doses of Pfs230D1-EPA/Alhydrogel^®^ (Clinicaltrials.gov NCT02334462) to identify functional monoclonal antibodies elicited in response to Pfs230. All samples were chosen from subjects with high serum transmission-reducing activity (TRA), measured by the ability of serum antibodies from immunized subjects to reduce the number of oocysts that develop in mosquitoes fed on in vitro cultured *P. falciparum* gametocytes (Supplementary Table 1).

We obtained 272 VH and 351 VL sequences of B cell receptor (BCR) from Pfs230D1-specific single memory B cells from vaccinees via amplification and sequencing of the V(D)J region (Supplementary Fig. 4). When analyzing V gene usage of the BCR sequences, 87.5% of the subjects presented Pfs230D1-specific memory B cells using kappa chains derived from IGKV4-1 (Supplementary Fig. 3e). This light chain gene has also been identified in sequences of functional human mAbs obtained in response to other *Plasmodium* antigens^7,8,10,11^. For the heavy chain, IGHV1-69 was the most commonly identified gene and was detected in 100% (8/8) of vaccinees (Supplementary Fig. 3f). IGHV1-69 is one of the most polymorphic loci of the IGHV gene cluster^12^ and is frequently found in broadly neutralizing antibodies generated in response to influenza haemagglutinin^13,14^.

**Fig. 1:**
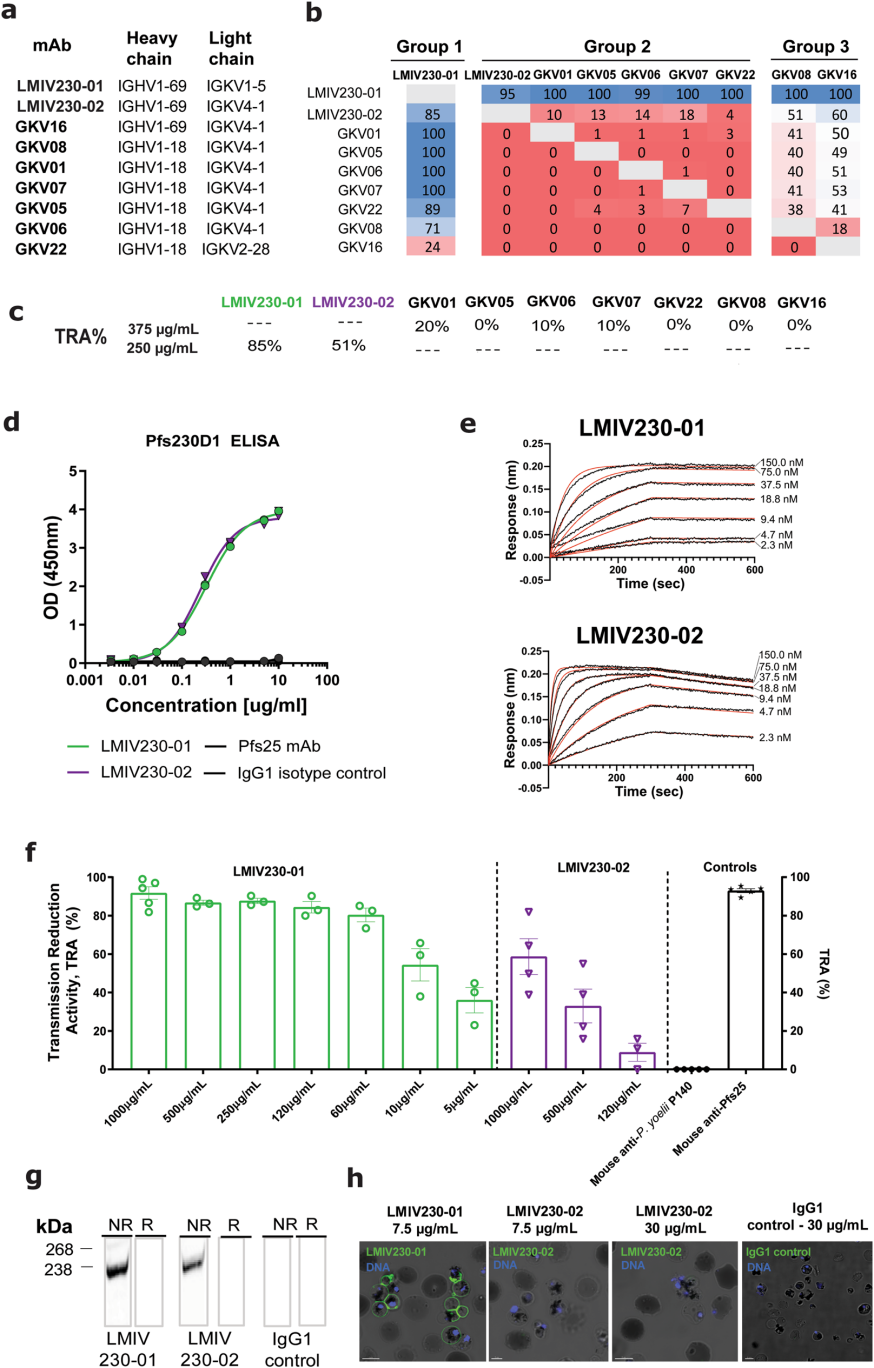
Human recombinant mAbs were generated from Pfs230D1-specific single memory B cells of Malian adults vaccinated with the Pfs230D1-EPA/Alhydrogel^®^ TBV. **a** VH and VL genes corresponding to each mAb. LMIV230-01 and LMIV230-02 sequences originate from the IGHV1-69 heavy chain gene but utilize different kappa chain genes. Complete V gene usage determined in Pfs230-specific memory B cells is described in Supplementary Fig. 3e, f. **b** Epitope binning of human anti-Pfs230D1 scFvs. The primary binding scFv is listed on the left and the competing scFv is listed on the top. Reported scores are a percentage of total binding of that antibody in the absence of a competitor scFv. Values greater that 50% display low amounts of competition, while values lower than 50% exhibit greater competition. Any experiment with >100% binding was given a score of 100, while negative values were given a score of 0. Potential epitope bins are grouped and labeled above the table. **c** Functional activity of each mAb, assessed by Standard Membrane Feeding Assay (SMFA) and measured as the % reduction (versus control mAb) in the number of *P. falciparum* NF54 oocysts in midguts of *Anopheles* mosquitoes (“TRA”). **d** LMIV230-01 and LMIV230-02 mAbs bound similarly to Pfs230D1 and (**e**) show high affinity to recombinant Pfs230D1 (Supplementary Fig. 5, Supplementary Table 2). **f** LMIV230**-**01 reduces *P. falciparum* NF54 oocyst numbers by 91.7% at 1000 µg/mL, 86.7% at 500 µg/mL, 87.7% at 250 µg/mL, and 80.3% at 60 µg/mL, while LMIV230-02 displays only modest activity with 58.7% reduction at the maximum concentration of 1000 µg/mL, in SMFA. At least three biological replicates were performed for each concentration of mAb, in the presence of intact serum containing human complement. *N* ≥ 20 mosquitos per assay. Average oocyst numbers in the control mosquitoes (fed with mouse IgG1 mAb targeting *P. yoelii* P140 protein) for each experiment were: exp. 1 = 29.73; exp.2 = 7.18; exp. 3 = 57.86; exp. 4 = 36.41; exp. 5 = 51.71, exp. 6 = 4.55; exp. 7 = 62.35; exp. 8 = 20.50, exp.9 = 8.71, exp 10 = 18.05, exp. 11 = 5.86. Negative oocyst reduction values were set to zero. Human isotype IgG1 and US human serum pool were used as additional negative controls (Supplementary Fig. 6b). Values are shown as mean ± s.e.m. **g** LMIV230-01 and LMIV230-02 bind to non-reduced (NR) protein extract of *P. falciparum* NF54 gametes purified on Nycodenz after 2 h in exflagellation medium. **h** LMIV230-01 binds to gametes at 7.5 µg/mL while LMIV230-02 does not bind at 7.5 µg/mL. or 30 µg/mL. Both mAbs were labeled with Alexa Fluor 488. Scale bars: 5 µM. This experiment was performed in triplicate. Source data are provided as a Source Data file.

Nine pairs of BCR sequences were chosen for expression of fully human Pfs230D1 IgG1 antibodies by assessing whether the CDR3 sequences were shared between sorted B cells. This approach identifies identical sequences in multiple B cells from the same subject, indicating that they have been activated in response to vaccination. These nine pairs (Fig. 1a) represented distinct heavy and light chain germline genes with an overabundance of IGHV1-18 (*N* = 6), IGHV1-69 (*N* = 3), and IGKV4-1 (*N* = 7). The resulting recombinant antibodies bound to Pfs230D1 antigen (Fig. 1d, e; Supplementary Fig. 5). Competitive epitope binning of the nine mAbs suggested they bind to three largely distinct group of epitopes in Pfs230D1 (Fig. 1b). LMIV230-01 forms a distinct group (Group 1) and has potent transmission-reducing activity (Fig. 1b, c). The remaining mAbs with low or no transmission-reducing activity form two additional epitope groups, Group 2 and 3 (Fig. 1c). We therefore focused most of our subsequent analyses on LMIV230-01, and to a lesser extent, LMIV230-02 which demonstrated low functional activity.

### LMIV230-01 and LMIV230-02 bind recombinant and native protein, but only LMIV230-01 mAb binds live gametes

Both LMIV230-01 and -02 bound to Pfs230D1 recombinant protein (Fig. 1d) with Kd values in sub-nanomolar range (Fig. 1e, Supplementary Fig. 5, Supplementary Table 2). We confirmed the two mAbs bind to distinct epitopes using competition ELISA (Supplementary Fig. 6d) consistent with the epitope binning results (Fig. 1b). Despite their shared use of IGHV1-69 (Supplementary Fig. 7), LMIV230-01 and LMIV230-02 displayed numerous differences in their heavy chain CDRs (Supplementary Table 7, Supplementary Fig. 13), consistent with their recognition of distinct epitopes.

Although both mAbs had a similar affinity to Pfs230D1, they differed in their functional activity as measured by SMFA. LMIV230-01 reduced *P. falciparum* oocyst burden in mosquitoes in a dose-dependent manner with 91.7% TRA at a concentration of 1000 µg/mL (Fig. 1f). Importantly, 80.3% TRA was retained at 60 µg/mL. LMIV230-02 reduced oocyst burden by 58.7% at a concentration of 1000 µg/mL and activity was lost at 250 µg/mL. As previously reported, TRA values higher than 80% are highly reproducible across independent experiments^15,16^. Combining the two antibodies did not increase their overall activity: TRA values were not statistically different when 500 µg of LMIV230-02 was combined with 10 µg of LMIV230-01 (TRA = 58.7%) versus 10 µg of LMIV230-01 alone (TRA = 52.5%) in mosquito feeding assays (Supplementary Fig. 6e).

To understand the differences in functional activity between the two mAbs, we assessed binding of LMIV230-01 and 02 to the native Pfs230 by Western blot and confocal microscopy. Both mAbs reacted to the protein extract of parasites and were sensitive to the reduction of the two disulfide bonds, suggesting the presence of conformational epitopes (Fig. 1g, Supplementary Fig. 6c). Additionally, LMIV230-01 labeled the surface of live *P. falciparum* gametes purified 2 h post-exflagellation, while LMIV230-02 did not (Fig. 1h). This suggests that the LMIV230-02 epitope is not completely accessible on the surface-displayed native protein, possibly due to structural limitations imposed by the multi-domain protein Pfs230, as has been seen for other proteins^17,18^ including another 6-Cys TBV candidate^19^.

**Fig. 2:**
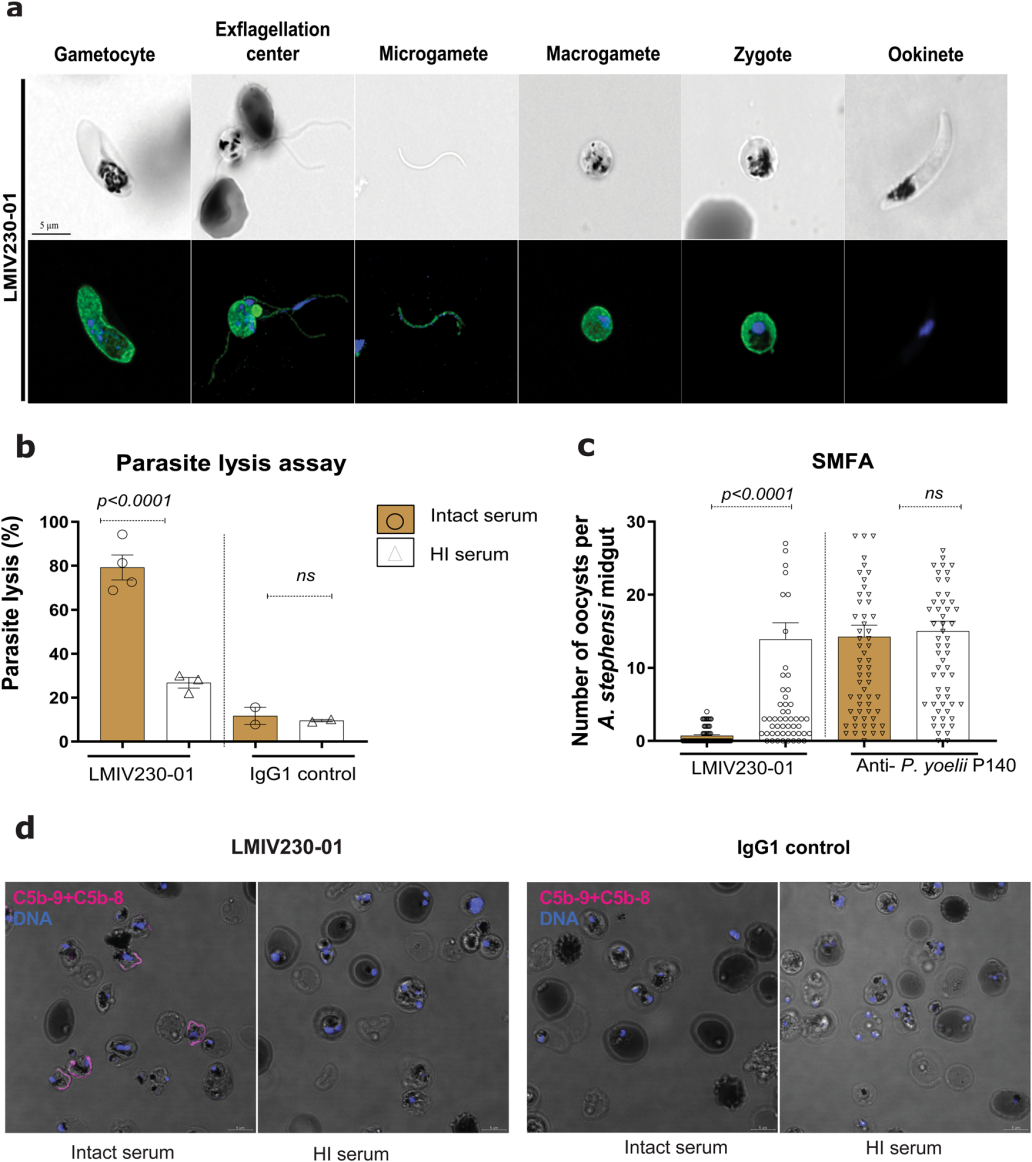
LMIV230-01 binds to multiple parasite stages and its activity is complement-dependent. **a** LMIV230-01 binds to permeabilized gametocytes, gametes, and zygotes and does not bind to ookinetes. Parasites were fixed and permeabilized, and 7.5 µg/mL of antibody was used to stain the different parasite stages. Scale bars: 5 µM. This experiment was performed in duplicate. **b** In vitro parasite lysis by LMIV230-01 is complement-dependent. Samples were tested in two independent assays, using two different parasite cultures. Data for LMIV230-02 mAb are shown in Supplementary Fig. 8a**. c** Functional activity of LMIV230-01 is also complement-dependent in vivo (SMFA with mosquitoes). Data from three independent SMFA assays. *N* ≥ 20 mosquitos per assay. Data for LMIV230-02 mAb are shown in Supplementary Fig. 8b. Oocyst averages in the control mosquitoes (fed with IgG1 targeting *P. yoelli* P140) for each of the experiments were: exp. 1 = 4.55; exp. 2 = 20.50, exp. 3 = 5.86. Data obtained from mosquitoes fed with LMIV230-01 at 1000 µg/mL with intact sera were also used to generate Fig. 1f. Values are shown as mean ± s.e.m. One-Way ANOVA and Tukey’s multiple comparisons test were used to compare the different groups. **d** Live imaging of *P. falciparum* NF54 female gametes incubated with LMIV230-01 in the presence of intact serum from a healthy donor revealed surface-deposited MAC (membrane attack complex) using anti-C5b-9 + C5b-8 antibody (magenta color). MAC deposition was not detected in the presence of heat-inactivated (HI) serum. Scale bars: 5 µM. This experiment was performed in triplicate. Source data are provided as a Source Data file.

LMIV230-01 bound to fixed parasites in numerous developmental stages including gametocytes, exflagellation centers, microgametes, macrogametes, and round forms (zygotes) collected 4 h after mosquito feeding. As expected, the mAb did not bind to post-fertilization ookinete stage parasites obtained 24 h after the mosquito bloodmeal (Fig. 2a).

### LMIV230-01 activity is dependent on human complement

Pfs230 antibody activity depends on complement fixation to lyse *P. falciparum*^20^. To test whether the activity of LMIV230-01 was dependent on activation of the complement system, we incubated parasites with LMIV230-01 in the presence of intact or heat-inactivated sera from US donors then measured lysis of gametes (Fig. 2b) as well as transmission of parasites fed to mosquitoes after treatment under the same conditions (Fig. 2c). Functional activity of LMIV230-01 to lyse gametes and block oocyst formation in mosquitoes was substantially reduced in the heat-inactivated sera (Fig. 2b, c), indicating complement-dependency. Activation of complement leads to the formation of the membrane attack complex (MAC), an assembly of the complement molecules C5b, C6, C7, C8, and C9^21,22^ on the parasite surface. Using an antibody that recognizes assembled MAC, we demonstrated complement fixation on the surface of live *P. falciparum* gametes that were incubated with LMIV230-01 in the presence of intact but not heat-inactivated serum (Fig. 2d and Supplementary Fig. 8c).

### LMIV230-01 binds to a large and conserved epitope in Pfs230D1

**Fig. 3:**
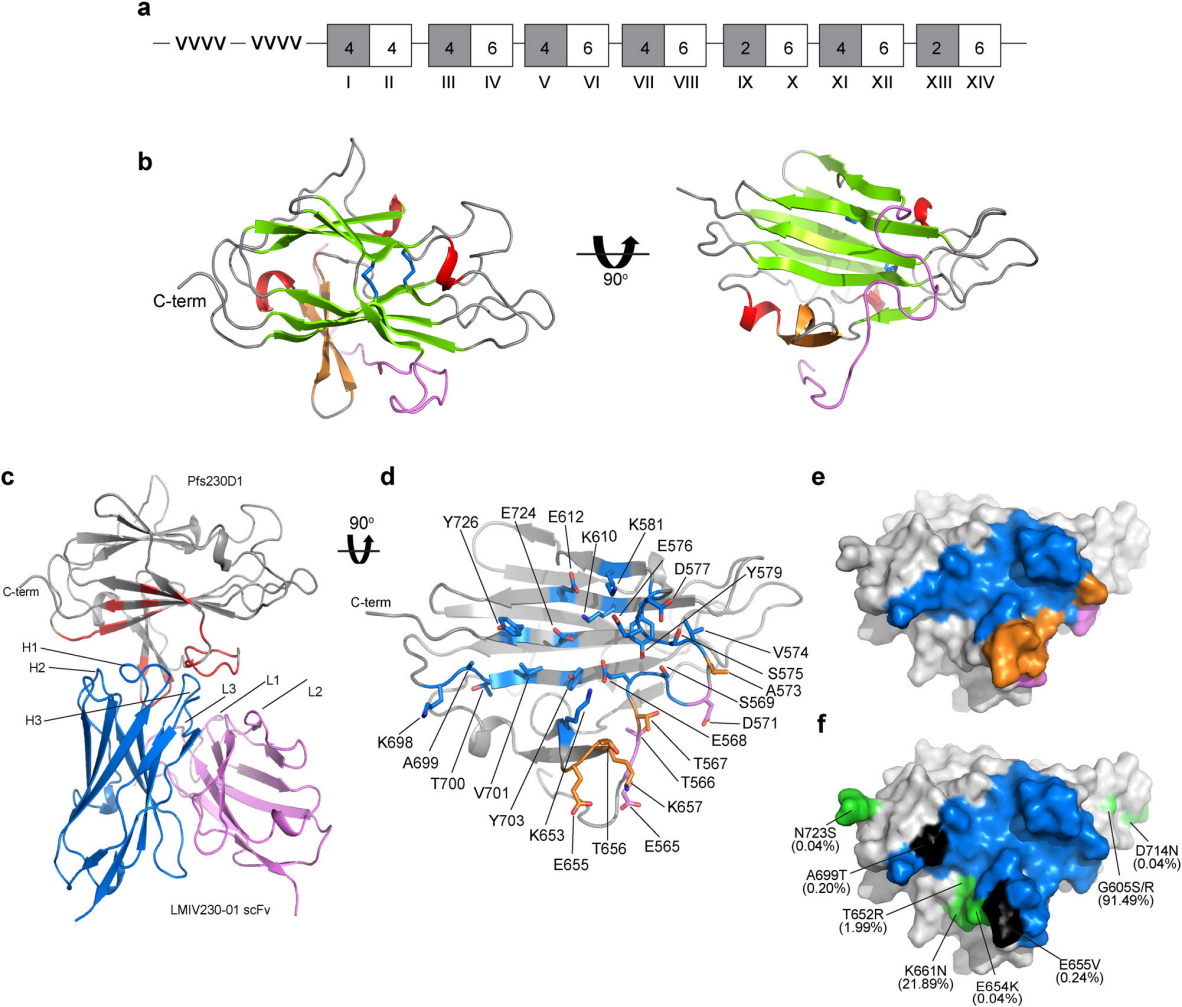
Structural definition of LMIV230–01 scFv epitope in Pfs230D1. **a** Domain organization of Pfs230D1. A-Type (gray) and B-Type (white) 6-Cys domains are shown in boxes. Numbers inside the boxes indicate the number of conserved cysteines. Domain numbers are labeled in Roman numerals. -vvvv- indicates a highly repetitive region that is cleaved from the mature protein. **b** Structure of Pfs230D1. N-terminal loop in violet, beta-sandwich in green, beta strands in orange, helices in red, loops in gray and disulfide bonds in blue sticks. **c** Overall structure and epitope for the Pfs230D1–LMIV230–01 scFv complex. Pfs230D1 in gray; LMIV230–01 scFv heavy chain in blue; LMIV230–01 scFv light chain in violet; epitope in red. **d** Orthogonal detailed view of the Pfs230D1 epitope for LMIV230–01 scFv. Pfs230D1 in gray ribbon; residues contacted by LMIV230–01 scFv heavy chain, light chain and both are highlighted in blue, violet, and orange, respectively. **e** Surface representation of the epitope on Pfs230D1. The orientation and the color scheme are the same as in (**b**). **f** Polymorphisms in Pfs230 mapped onto the surface of Pfs230D1. Orientation as in (**c**). The LMIV230–01 scFv binding epitope in blue, polymorphic residues are in green, polymorphic residues within the epitope are in black. The frequency of the polymorphisms is in parentheses.

The potent transmission-blocking activity and distinct epitope reactivity of LMIV230-01 against the parasite prompted a structural analysis to identify the epitope and further inform biology and structural vaccinology. We solved the crystal structure of Pfs230D1 in complex with a single chain variable fragment (scFv) derived from mAb LMIV230-01 at 2.0 Å resolution (Fig. 3, Supplementary Table 3 and Supplementary Fig. 9). The structure of Pfs230D1 is composed of a mix of beta strands organized in a five-on-four sandwich (Fig. 3b). Structural homology search using the DALI server^23^ revealed that Pfs230D1 closely resembles the 6-Cys domains from Pf12-D2, Pf41-D2, and Pfs48/45-D3 (Supplementary Fig. 10). However, four cysteines in Pfs230D1 are paired (C593–C611, C626–C706) (Supplementary Fig. 11) in contrast to the other 6-cys domains that have six cysteines as three disulfide bridges. In addition, the Pfs230D1 antigen contains a long N-terminal extension (residues 557–579) beyond the 6-Cys domain core that packs against the 6-Cys core (Fig. 3b).

All six complementarity-determining regions (CDRs) of the antibody contact Pfs230D1 **(**Fig. 3c, d and Supplementary Table 4**)**, forming an extensive buried surface area of the epitope (1047 Å^2^) on Pfs230D1 that is primarily contributed by the heavy chain (750 Å^2^) and less by the light chain (297 Å^2^). The discontinuous conformational epitope on Pfs230D1 comprises contact sites on five beta strands and the N-terminal loop that is unique to Pfs230D1. The antigen–antibody complex predominantly stabilized by hydrophobic interactions is mediated by E565, T566, T567, E568, G570, D571, A573, V574, S575, D577, Y579, K581, K610, E612, T656, K698, A699, T700, V701, Y703, E724, and Y726 in Pfs230D1. Five residues in Pfs230D1 (S569, E576, K653, E655, and K657) form hydrogen bonds or salt bridges with the antibody (Fig. 3d, e and Supplementary Table 4). The conformational transmission-blocking epitope contains a major segment of the Pfs230D1-specific N-terminal region and this contributes to the specificity of LMIV230-01 towards domain 1 of Pfs230.

Analysis of 2512 Pfs230D1 sequences (from MalariaGen, representing diversity across Africa and Asia) revealed that the major polymorphisms are outside the binding epitope for LMIV230-01 (Fig. 3e, f and Supplementary Table 5). The most common polymorphism observed in 91.49% of the sequences is G605S/R, and this residue is located outside the binding epitope. Two medium frequency polymorphisms T652R (1.99%) and K661N (21.89%) are located adjacent to the epitope. Two low-frequency polymorphisms E655V (0.24%) and A699T (0.20%) are located within the binding epitope.

Binding affinity measurements of five polymorphisms that are located within or adjacent to the epitope showed the mutants did not negatively affect binding affinity as compared to the wild-type Pfs230D1 protein (Supplementary Table 6, Supplementary Fig. 4c–g). Only A699T resulted in increased affinity, which can be explained by the threonine replacement creating additional hydrophobic contacts upon modeling the change into the structure (Supplementary Fig. 12). These results indicate that LMIV230-01 should bind >99% of known Pfs230 variants suggesting a broadly conserved epitope.

A single N-glycosylation mutation (N585Q) was introduced in Pfs230D1 to prevent aberrant glycosylation in the *Pichia pastoris* expression system and derive the protein immunogen preferred for vaccination (Supplementary Fig. 10). This mutation is located far from the LMIV230-01 binding epitope and should not affect the ability of Pfs230D1 to induce transmission-blocking mAbs similar to LMIV230-01. Finally, LMIV230-01 has no insertions or deletions in the CDRs as compared to germline sequences, suggesting a reliable route to antibody production and maturation upon vaccination (Supplementary Fig. 11).

### LMIV230-01 is functional against heterologous parasites

**Fig. 4:**
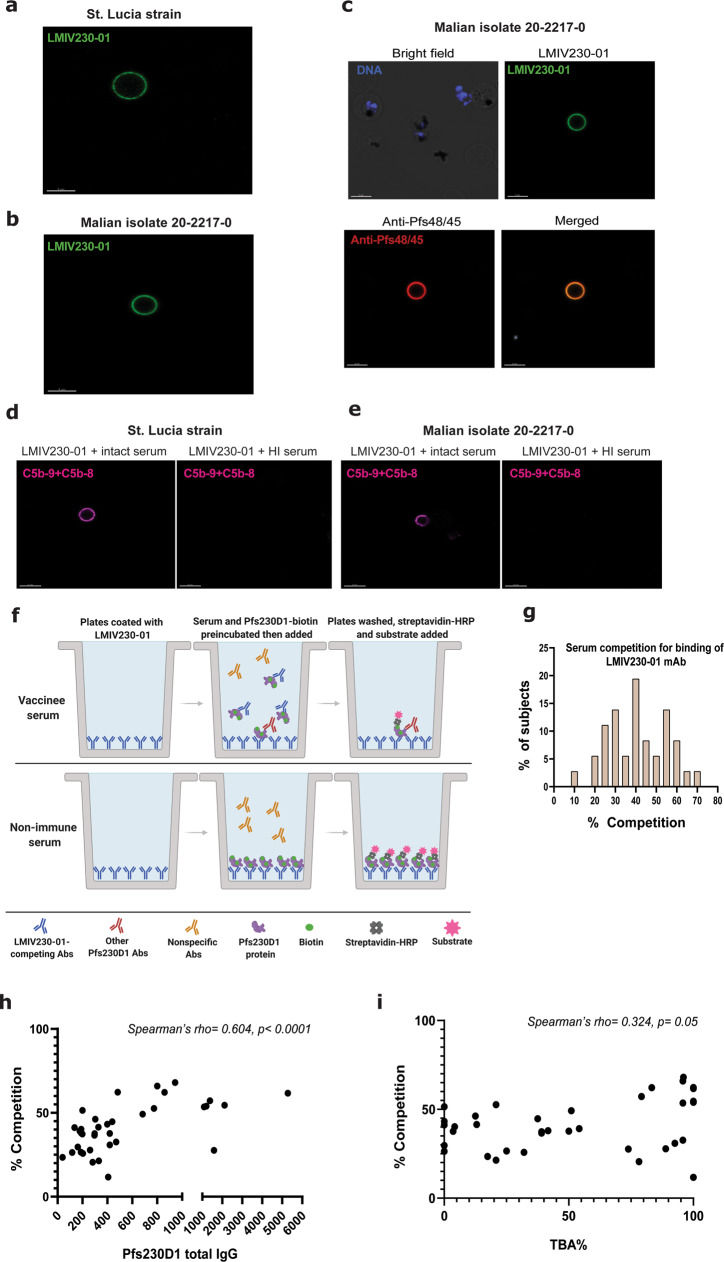
LMIV230-01 binds to heterologous *P. falciparum* strains and antisera from Pfs230D1 vaccinees vary widely in levels of antibody that compete with LMIV230-01 for binding. **a** LMIV230-01 bound to gametes of *St. Lucia* parasite strain and **b** of an isolate obtained from a Malian adult and adapted to culture. **c** Murine anti-48/45 mAb confirms formation of gametes by Malian isolate and its signal colocalizes with LMIV230-01. “Merged” refers to combination of green and red channels. **d** Membrane attack complex forms on gametes of St. Lucia strain and (**e**) of a Malian isolate incubated with LMIV230-01 in the presence of intact but not heat-inactivated serum. All imaging experiments shown in this figure were performed in duplicate. Scale bars for all images in this panel: 5 µM. **f** Cartoon schematizing LMIV230-01 competition ELISA assay. **g** Distribution of serum antibody levels that compete with LMIV230-01 for binding to Pfs230D1 in 36 subjects who received Pfs230D1-EPA vaccine. Values displayed represent mean from three independent experiments. **h** Relationship of LMIV230-01-competing antibody levels to total Pfs230D1 antibody titers, or (**i**) to serum functional activity (TBA, transmission-blocking activity) measured by SMFA. Source data are provided as a Source Data file.

To assess whether LMIV230-01 would also bind to other *P. falciparum* strains, we prepared gametocytes from a culture-adapted Malian isolate^24^ and from the St. Lucia strain (originally from El Salvador)^25^. LMIV230-01 labeled in vitro induced gametes from both strains (Fig. 4a). Induction of gamete stage from the newly characterized Malian isolate was confirmed using a murine anti-Pfs48/45 mAb (Fig. 4b). LMIV230-01 fixed complement on the gamete surface of both strains, confirming that the antibody is functional against heterologous parasites (Fig. 4c, d). DNA sequencing revealed the mutation G605S in the Malian isolate and the G605S and the K661N mutations in the St. Lucia line, representing the most common polymorphisms (Supplementary Fig. 14).

### Human antisera commonly compete with LMIV230-01 for binding to Pfs230D1 and competition correlates to blocking activity

To assess the abundance of antibodies that share paratope specificity with LMIV230-01, we developed an ELISA assay to demonstrate the competition between post-vaccination sera (tested at a 1:2500 dilution) and LMIV230-01 for binding the vaccine antigen (Fig. 4f). Among subjects who received the vaccine, levels of competing antibody ranged from ~10–70% displacement of Pfs230D1 binding to LMIV230-01, with a normal distribution confirmed by Shapiro–Wilk test (*p* = 0.52) (Fig. 4g). Levels of competition correlated with total Pfs230D1 IgG titers in sera (Spearman’s rho = 0.604, *p* < 0.0001) (Fig. 4h).

Increasing levels of competing antibody also corresponded to serum functional activity measured by SMFA. Since serum TRA levels of vaccinees were high with minimal variability ranging from 95 to 100% (Supplementary Fig. 15), our primary correlation analysis used TBA (Transmission-Blocking Activity), which indicates the percent reduction in the proportion of infected mosquitoes, a stringent criteria for TBV activity generally seen only when TRA is very high. Correlation analyses showed that percent serum competition was related to TBA (Spearman’s rho= 0.324, *p* = 0.05) (Fig. 4i), suggesting that antibodies that compete for the LMIV230-01 epitope play a role in serum functional activity. This result, however, does not exclude the possible role of other antibodies (that do not compete with LMIV230-01) in mediating vaccine activity; notably, some sera with high TBA demonstrated low levels of LMIV230-01 competing antibodies.

Due to its complex domains and repeating motifs with numerous disulfide bonds, expression of full length Pfs230 has been difficult^26,27^. Preclinical studies of Pfs230 fragments have shown that immunization with recombinant domain 1 of Pfs230 (Pfs230D1), but not other domains, induces strong functional TRA in SMFA^3,26,28^. Altogether, our data confirm that Pfs230D1 vaccination can elicit strong transmission-blocking antibodies, capable of binding to gametocytes, gametes, and zygotes, and impairing fertilization in the mosquito. However, not all mAbs that bind Pfs230D1 block transmission and not all Pfs230D1 vaccine recipients develop high levels of antibody that compete with LMIV230-01 for binding antigen. Only one mAb (LMIV230-01) out of nine examined in this study has high transmission-blocking activity.

The structural definition of the potent transmission-blocking epitope described here provides a rational basis to improve Pfs230D1-based TBVs, and the recombinant antibodies can also be developed as interventions. The structural findings will inform immunogen design to focus the immune response and to increase antibody titers to the LMIV230-01 epitope, as one approach to enhance vaccine activity. Immunogen improvements can include designs that expose transmission-blocking epitopes while shielding or eliminating non-functional epitopes^29,30^. This study also enables the development of assays that incorporate LMIV230-01 or mutant Pfs230D1 that lack the LMIV230-01 epitope to evaluate vaccine-induced or naturally acquired immune responses. These assays can accelerate the assessment and hence the development of optimal Pfs230D1 immunogens that enhance LMIV230-01-like responses over non-functional responses.

## Methods

### Human ethics statement

This study was approved by the ethics review boards from the Faculté de Médecine de Pharmacie et d’OdontoStomatologie (FMPOS), Bamako, Mali, and the US National Institute of Allergy and Infectious Diseases (NIH, Bethesda, MD, USA), as well as the Mali national regulatory authority. Written informed consent was obtained from study participants. The clinical trial was registered in clinicaltrials.gov (NCT02334462) and is published elsewhere^31^.

### Human immunization and samples collection

Malian adults were vaccinated with four doses of 40 µg of Pfs230D1-EPA/Alhydrogel® at days 0, 28, 168, and 540. Sera were obtained from eight participants, 14 days after the 4th dose (day 554) to measure serum transmission reduction activity (TRA) by standard membrane feeding assay (SMFA) as previously described^32^. PBMCs (peripheral blood mononuclear cells, 5 million cells per tube on average) were prepared from the same participants for isolation of Pfs230D1-specific single memory B cells.

### Statistical analyses

One-Way ANOVA followed by multiple comparisons was used to compare data from different groups. Results in the graphs are displayed as mean + SEM.

### Expression of Pfs230D1

Recombinant domain 1 of Pfs230 protein (3D7, accession number XP_001349600.1) was expressed in *Pichia pastoris* as previously described^26^. Briefly, Pfs230D1 was secreted during the methanol induction phase into a chemically defined media and recovered from the fermentation broth following centrifugation, microfiltration, ultrafiltration, and diafiltration. A concentrated Pfs230D1 feed stock was purified by mixed-mode, ion-exchange, and size exclusion column chromatography. The bulk protein was stored at −80 °C prior to use.

### Pfs230D1 B cell tetramer construction

A tetramer for selection and sorting of Pfs230D1-specific B cells was generated as previously described^33,34^. Briefly, recombinant Pfs230D1 was chemically biotinylated using EZ-Link Sulfo-NHS-LC-Biotin (Thermo Fisher Scientific, Waltham, USA). The biotinylated protein was then tetramerized with streptavidin labeled with PE (Prozyme, Hayward, USA). A decoy tetramer that excludes non-Pfs230D1 specific B cells was generated by conjugating BSA to biotin and streptavidin-PE as described above, and later conjugating the tetramer with Alexa Fluor™ 594 using DyLight 594 Protein Labelling Kit (Thermo Fisher Scientific, Waltham, USA).

### Enrichment of Pfs230-specific cells in PBMCs

PBMCs were thawed in 37 °C water bath, resuspended in complete RPMI and washed with PBS. 1 µM of BSA-decoy tetramer was added to 2 to 3 million PBMCs and incubated at room temperature for 5 min, protected from light. 1 µM Pfs230D1-PE tetramer at was added to the cells and then incubated at 4 °C for 20 min. Cells were washed with PBS containing 10% FBS and incubated with 25 µL of anti-PE magnetic beads (Miltenyi Biotech, Bergisch Gladbach, Germany) for 25 min. Four mL of PBS were added to the solution, which was then passed over magnetized LS columns for elution of cell suspension enriched for Pfs230D1-PE cells.

### Flow cytometry and sorting

After enrichment with Pfs230D1 tetramer, human cells were stained with the following surface-conjugated antibodies: CD3 (UCHT1), CD14 (M5E2), CD56 (HCD56) Alexa Fluor 700, CD19 APC-CY7 (HIB19) CD20 PE-CY7 (2H7), and CD27 APC (LG.3A10) purchased from Biolegend (San Diego, USA).

A gating strategy was performed first for doublet discrimination, then singlet cells were selected for exclusion of non-B cells using CD3, CD14, and CD56 markers. Lymphocytes were gated for CD19+CD20+. Further discrimination with CD27+ allowed gating for memory B cells. Pfs230D1-specific B cells were gated using PE, and non-Pfs230D1 cells excluded using CF594, the fluorochrome used in the decoy BSA tetramer. Analysis was performed in FACSAria™ II instrument (Fluorescence-activated cell sorting, BD Biosciences, San Jose, USA) with blue, red, and violet lasers. Pfs230D1-specific memory B cells were analyzed according to the fluorescence staining profile described above and sorted directly into 96-well PCR plates using a 100 µm nozzle. After sorting, plates were immediately centrifuged at 1278 × *g* for 30 s, transported on dry ice and stored at −80 °C.

### BCR sequencing of Pfs230D1-specific single memory B cells

Amplification of BCR heavy and light chains from single sorted cells was performed by iRepertoire Inc. (Huntsville, AL, USA). RT-PCR1 was performed with nested, multiplex primers covering both heavy, kappa, and lambda loci, and including partial Illumina adaptors. Included on the reverse primer was an in-line 6 nucleotides barcode, which served as a plate identifier so that multiple 96-well plates could be multiplexed in the same sequencing flow cell. After RT-PCR1, the first round PCR1 products were rescued using SPRISelect Beads (Beckman Coulter, Brea, USA). A second PCR was performed with dual-indexed primers that complete the sequencing adaptors introduced during PCR1 and provide plate positional information for the sequenced products. Sequencing was performed using the Illumina MiSeq v2 500-cycle kit with 250 paired-end reads.

Raw data were demultiplexed by Illumina dual indices and the 6-nt internal plate barcode information for each well of the 96-well PCR plates. Data were analyzed using the previously described iRmap program^35^. Reads were trimmed according to their base qualities with a 2-base sliding window. If the quality value in this window was lower than 20, the sequence stretch from the window to the 3′ end was trimmed from the original read. Trimmed pair-end reads were joined together through overlapping alignment with a modified Needleman-Wunsch algorithm. If paired forward and reverse reads in the overlapping region were not perfectly matched, both forward and reverse reads were thrown out without further consideration. The merged reads were mapped using a Smith-Waterman algorithm to germline V, D, J, and C reference sequences downloaded from the IMGT web site^36^. To define the CDR3 region, the position of CDR3 boundaries of reference sequences from the IMGT database was migrated onto reads through mapping results, and the resulting CDR3 regions were extracted and translated into amino acids. The data for each chain of the receptor pair begins from within the beginning of framework 1 and extends to the beginning of the C-region (including the isotype).

### BCR sequence analysis

BCR sequence data were submitted to IMGT/HighV-QUEST for annotation of V, D, and J gene usage, determination of mutation from germline, CDR sequence extraction, and translation^37^. Clustering of related sequences was performed using a previously described method that groups together sequences arising from the same clonal expansion^38^. To be considered part of the same cluster, sequences were required to have the same V and J gene usage and CDR3 length for both chains, and no more than 10% different nucleotides in the combined CDR3.

Germline sequences were reconstructed using the CreateGermlines function of the Change-O software package^39^. Phylogenetic lineages were created from sequence clusters using the Alakazam R package^37,40^ and rooted to the germline. Sequence alignments were visualized using the msa R package^41^ and graphs plotted using the ggplot2 R package^42^.

### Pfs230D1 mAbs expression

We chose nine BCR sequences from four different subjects for mAb expression based on evidence of clonal expansion (presence of identical sequences in multiple B cells from the same subject), as previously described for selection of T cell receptors^43^.

For LMIV230-01 ad LMIV230-02, PCR products (obtained from iRepertoire Inc.) containing the 3′ end of framework 1 through the J region of heavy chain variable region were used as template. To restore the 5′ end of framework one, PCR was performed using oligos MBfor1 and MBrev1. The product of this PCR was then used to complete framework region one through amplification using MBfor2 and MBrev1. This second PCR product was then reamplified using MBfornodig1 and MBrevnodig1 to recover the entire variable region while adding sequence compatible with the IL-2 leader sequence at the 5′ end and CH1 3′. Kappa light chains were restored similarly using oligos MBkapfor1 and MBkaprev1, MBkapfor2 and MBkaprev2, and MBkapfornodig1 and MBkaprevnodig1 (Supplementary Table 8). Amplifications were performed using the Q5 polymerase (New England Biolabs, Ipswich, USA) by dissociation at 98 °C for 30 s, annealing at 61 °C for 30 s, and extension at 72 °C for 30 s. PCR products cut from 2% agarose gels were ligated into the PFUSEss-CHIg-hG1 (Invivogen) previously digested using *EcoRI* and *NheI* or PFUSE2ss-CLIg-hk (*EcoRI* and *BsiWI*) vectors (InVivogen) using the In-Fusion HD cloning kit from Clontech (part number 639649) to create plasmids pMB001 (LMIV-230-hF-001 heavy), pMB002 (LMIV-230-hF-001 kappa), pMB003 (LMIV-230-hF-002 heavy) and pMB004 (LMIV-230-hF-002 kappa). Transfections of HEK 293 F cells using freestyle 293 media (Invitrogen part no 12338026), and 293 fectin (Invitrogen part no 12347019) were performed according to the manufacturer’s protocol. Antibodies were purified on a 5 mL HiTrap Protein A column (Qiagen). Human IgG1 (with heavy and kappa chains) isotype control was purchased from Creative Biolabs (Shirley, USA). The other seven IgG1 mAbs were expressed in HEK293 cells by LakePharma, Inc (San Carlos, USA), and characterized to meet the manufacturer’s requirements for purity, concentration, and endotoxin levels.

### Enzyme-linked immunosorbent assay (ELISA) for Pfs230D1

mAb responses against recombinant Pfs230D1 were measured using ELISA. Immulon^®^ 4HBX plates were coated with 1ug/well of recombinant Pfs230D1. Plates were incubated overnight at 4 °C and blocked with 320 µL of buffer containing 5% skim milk powder in Tris-buffered saline for 2 h at RT. Plates were washed with Tween-TBS. Samples (dilution 1:500) were added to Pfs230D1-coated wells, in triplicate, and incubated for 2 h at RT. Plates were washed, then 100 µL of alkaline phosphatase labeled goat anti-human IgG were added. Plates were incubated for 2 h at RT and washed. A colorimetric substrate, p-nitrophenyl phosphate (Sigma, St. Louis, USA) was added and plates were read at absorbances of 450 nm and 550 nm on a multi-well reader (Molecular Devices, San Jose, USA).

### Competition ELISA between LMIV230-01 and LMIV230-02

LMIV230-01 mAb was conjugated to HRP using the EZ-Link plus activated peroxidase kit (VWR). Each well was coated with 50 ng of Pfs230D1 in 100 µL of PBS for 1.5 h and blocked with 3% milk. Unconjugated LMIV230-02 mAb was titrated from 2000 to 1 ng/well and incubated 2.5 h at room temperature in PBS with 3% milk. Sample was removed, and conjugated LMIV230-01 was added at 1:250 dilution. After 1 h, wells were washed four times with 140 µL of phosphate buffered saline (PBS), plates were developed using TMB one step substrate (VWR) and read at absorbances of 450 nm and 550 nm on a multi-well reader.

### *P. falciparum* stages preparation

#### Gametocytes

Stage V gametocytes were recovered from *P. falciparum* culture and immediately centrifuged at 500 × *g* to be further used, either stained for live imaging or fixed with 4% paraformaldehyde and 0.08% glutaraldehyde in PBS for 30 min. Cells were then used for immunofluorescence as described below.

#### Female gametes

Ten milliliters of a gametocyte stage V culture were centrifuged at 2000 rpm for 5 min and added to an exflagellation medium containing 900 µL of RPMI, 100 µL of PBS, and 1 µL of xanthurenic acid, then left for 2 h at room temperature. Cells were resuspended in 5 mL of RPMI and overlaid on a 15 mL cushion of Nycodenz gradient (16%, 11%, and 6%), then centrifuged at 7000 × *g* for 30 min with no break. Parasites located in the interface between 6% and 11% were collected into 50 mL of RPMI and spun down at 2000 × *g* for 10 min.

#### Male gametes and exflagellation centers

Male gametes and exflagellation centers were obtained following the same protocol described to activate female gametes. However, the parasites were recovered after 12 min of activation instead of 2 h.

#### Round form (zygotes) and ookinetes

Mosquito midguts were dissected in PBS either 4 h or 24 h post blood meal for collection of zygotes and ookinetes, respectively. The collected parasites were then used for immunofluorescence assays.

### Culture and genotyping of Malian isolate of *P. falciparum*

Malian isolate 20-2217-0 was obtained from a blood sample collected in Ouélessébougou (Mali) through a previously described immune-epidemiology study^24^, and lab-adapted by maintaining the culture over three weeks. Isolate 20-2217-0 was grown in RPMI 1640 supplemented with L-Glutamine + 25 mM Hepes + 50 ug/ml Hypoxanthine (K.D. Medical, Columbia MD), 5 g AlbuMAXII, Lipid Rich BSA (Gibco, Gaithersburg, MD), 2 g Dextrose, Anhydrous (Thermo Fisher Scientific, Waltham, USA), 2.25 g Sodium bicarbonate (Mallinkrodt, Staines-upon-Thames, UK) and human sera pool. Gamete stage was induced in vitro as previously described and Pfs230 domain 1 was amplified from gametes using the following primers, ATTTATAGAAGGGGGTGAAGGAGATGATG and TTAGGAAATAGAATACCACCTATATCTCCGC, and sequenced to identify polymorphisms.

### Immunofluorescence of fixed parasites

Approximately 2.5 × 10^5^ parasites were resuspended in 100 µL PBS and adhered to slides covered with poly-l-lysine for 30 min at room temperature. The cells were fixed in 4% paraformaldehyde and 0.08% glutaraldehyde diluted in PBS, then washed twice in PBS. The gametocytes were permeabilized with 0.1% Triton X-100/PBS for 10 min at room temperature and blocked with 2% BSA in PBS for 1 h at room temperature. Parasites were probed with 7.5 µg/mL of mAbs LMIV230-01, LMIV230-02, or human IgG1 isotype control in blocking buffer for 2 h at room temperature, then washed three times in PBS. Coverslips were mounted using Prolong Gold mounting media (Thermo Fisher Scientific, Waltham, USA) and slides were kept at 4 °C prior to visualization using a Leica SP8 confocal microscope (Leica, Wetzlar, Germany).

### Immunofluorescence of live parasites

2.5 × 10^5^ *P. falciparum* female gametes were incubated with 7.5 µg/mL of LMIV230-01, -02 or human IgG1 isotype control each mAb for 1 h at 37 °C. Parasites were then stained with Hoechst 33342 Solution (1:20000 dilution) for 5 min and washed with PBS. Prolong Live Antifade Reagent (Thermo Fisher Scientific, Waltham, USA) was added and parasites were kept at 37 °C for up to 2 h until imaging, performed using a Leica SP8 confocal microscope (Leica, Wetzlar, Germany).

### Gamete lysis by complement

2.5 × 10^5^ *P. falciparum* gametes in RPMI 1640 were aliquoted into Eppendorf tubes. Ten µL of nonimmune human serum were added to each experimental tube to serve as a source of complement proteins. In the control tubes, 10 µL of nonimmune human sera was added prior to heat-inactivation at 56 °C for 20 min. Twenty µL of each mAb at 1 mg/mL was added to the tubes and incubated at 37 °C for 30 min. After the incubation, 15 µL of the suspension was placed on microslides, and the number of lysed gametes was counted by DIC microscopy, using the 40x objective. Three biological replicates with two technical replicates each were performed.

### Deposition of the MAC

Formation of MAC and further activation of the complement system were assessed using Immunofluorescence of live female gamete parasites. Female gametocytes (prepared as above) were incubated with 10 µg/mL LMIV230-01 diluted in PBS. The suspension was incubated at 37 °C for 45 min with either 50 μL of intact sera or sera heat-inactivated at 56 °C, both obtained from a healthy US donor. During the incubation the tubes were gently mixed every 10 min to facilitate C5b-9 and C5b-8 deposition on cells. Two milliliters of cold PBS were used to stop the reaction and to wash the cells. The suspension was centrifuged at 500 × *g* for 5 min, and the pellet then incubated with 10ug/mL of the mAb anti-C5b-9 + C5b-8 (Abcam, ref ab66768) for 2 h on ice. Cells were washed with PBS, centrifuged at 500 × *g* for 5 min, stained with Hoechst 33342 Solution (Thermo Fisher Scientific, Waltham, USA) diluted at 1:20,000 for 8 min, and further washed with PBS. Cells were kept in parasite culture media during imaging, which was performed using a Leica SP8 confocal microscope equipped with an environmental chamber (Leica, Wetzlar, Germany). Quantification of mean fluorescence intensity was performed using the “Cell” feature in Imaris software (Bitplane, Zürich, Switzerland) to analyze individual parasites. IgG1 isotype control with heavy and kappa chains was purchased from Creative Biolabs (Shirley, NY).

### Western blot

1.3 × 10^4^ female gametes of *P. falciparum* were resuspended in 80 µL of 1× SDS loading buffer (Li-Cor). Forty microliters of resuspended gametes were further diluted into the same volume of 1× SDS loading buffer with or without 10% beta-mercaptoethanol (aiming protein reduction), for a final concentration of either 0% or 5%. Beta-mercaptoethanol and samples were boiled at 95 °C for 5 min. Seventeen microliters of lysate were loaded on 3–8% Tris-acetate gel (Thermo Fisher Scientific, Waltham, USA) and separated for 60 min at 150 volts. Proteins were blotted to a nitrocellulose membrane using the iBlot transfer apparatus (Thermo Fisher Scientific, Waltham, USA) and blocked overnight with 5% powdered dry milk in TBST. Antibodies were diluted to a final concentration of 5 µg/mL in 5% powdered dry milk in TBST. Blots were incubated with primary antibody for 60 min at room temperature and washed 3 times for 5 min with TBST. Secondary antibody was diluted 1:5000 into 5% powdered dry milk in TBST. Blots were incubated with secondary antibody IRDye 800CW Goat anti-Human IgG (Li-Cor, Lincoln, USA) for 60 min at room temperature, washed 3 times for 5 min with TBST, and then rinsed 5 times with TBS. Blots were imaged with the Li-Cor Odyssey inferred imager (Li-Cor, Lincoln, USA).

### Epitope binning using bio-layer interferometry (BLI)

Epitope binning experiments were conducted at 30 °C using Pfs230D1 and scFvs. Recombinant Pfs230D1 were biotinylated in vivo in Expi293 cells. All proteins were buffer exchanged using size exclusion columns into buffer 10 mM HEPES pH 7.4, 150 mM NaCl, 3 mM EDTA. Concentrated Pfs230D1 and scFvs were diluted with kinetic buffer HBS-EP+ buffer to 20 nM and 150 nM, respectively. Streptavidin biosensors were dipped into wells containing biotinylated Pfs230D1 for loading. After 60 s of baseline incubation, the sensors were dipped into wells containing the first scFv to saturate the binding to Pfs230D1. After 60 s of baseline incubation, the sensors were dipped into wells containing the second scFv (75 nM) for competitive binding. Binning data were analyzed using Octet DataAnalysis HT 11.1 software.

### Bio-layer Interferometry (BLI) of anti-Pfs230D1 mAbs binding to Pfs230D1 variants

Anti-Pfs230D1 mAbs LMIV230-01 and LMIV230-02, and Pfs230-D1 variants, were buffer exchanged using size exclusion columns into buffer 10 mM HEPES pH 7.4, 150 mM NaCl, 3 mM EDTA. All proteins were concentrated using Amicon concentrators (Millipore, Burlington, MA) with a 10 kDa molecular weight cut-off. mAbs were diluted in HBS-EP+ buffer (GE Healthcare) to a concentration of 100 nM. Pfs230-D1 was diluted in HBS-EP+ buffer to 150 nM and a two-fold dilution series in HBS-EP+ buffer created for both concentrations. Anti-Human Fc Capture biosensors (Fortebio, Fremont, USA) were used with an OctetRed96e system (Fortebio, Fremont, USA) to conduct Bio-Layer Interferometry (BLI) experiments of mAb binding to Pfs230D1. In the BLI assay, either mAb LMIV230-01 or LMIV230-02 were bound to the sensors and then dipped into the Pfs230D1 dilution series. Both the Association and Dissociation steps were 300 s long. Kinetic analysis of the BLI data was conducted using Octet Data Analysis HT 11.1 software (Fortebio, Fremont, USA) and used all concentrations of Pfs230D1 for the kinetic analysis.

### Standard membrane feeding assay

SMFA was performed as previously described^44,45^ to assess transmission-reducing activity (TRA) in infected mosquitoes. Percentage reduction of *P. falciparum* oocyst numbers was calculated 8 days after mosquito feeding with immune serum as compared to negative control (mouse anti *P. yoelii* P140 at 375 µg/mL). Additional negative controls were used to confirm the oocyst reduction activity of the Pfs230D1 mAbs (human IgG1 isotype at 1000 µg/mL and pooled sera from US malaria-naive donors). A mouse anti-Pfs25 mAb (4B7, at 175 µg/mL) was used as a positive control. Briefly, an in vitro 15-day culture of *P. falciparum* (NF54 line) containing stage V gametocytes was diluted with washed O + RBCs (Interstate Blood Bank, Memphis, USA) and an AB + serum pool (not heat-inactivated) from US malaria-naive subjects (Interstate Blood Bank, Memphis, USA) to final concentration of 0.07%–0.1% stage V gametocytes and 50% hematocrit. For each individual assay, 200 µL of diluted culture was mixed with 60 µL of mAb diluted in PBS. Samples were then fed to pre-starved (24–30 h) 3–8-day-old *Anopheles stephensi* (Nijmegen strain) mosquitoes using a Parafilm membrane on a mosquito feeder, kept warm with 40 °C circulating water. After feeding, mosquitoes were kept at 26 °C and 80% humidity conditions to allow parasites to develop. On Day 8 after the feed, mosquito midguts were dissected and stained with 0.05–0.1% mercurochrome solution in water for 20–30 min. Infectivity was measured by counting oocysts in at least 20 mosquitoes per sample. Each sample was tested in at least two independent SMFAs. For SMFA experiments performed to assess complement-dependent activity of the mAbs, sera were heat-inactivated at 56 °C before the addition of mAbs.

### Serum competition ELISA for LMIV230-01

A chemiluminescent-based ELISA was developed to measure serum inhibition of binding between LMIV230-01 and Pfs230D1. Purified LMIV230-01 was coated at 100 ng/well and blocked with 1% BSA in PBS. Biotinylated Pfs230D1 at 500 pg/ml was incubated with serum (diluted at 1:2500) for 1 h and added to the coated well. After 1 h, plates were washed 4× and Streptavidin-poly HRP (Thermo Fisher Scientific, Waltham, USA) was added at 1:20000. Following incubation for 1 h, plates were washed 4× and SuperSignal ELISA Femto Substrate (Thermo Fisher Scientific, Waltham, USA) was added. To determine concentration of inhibitory antibody, chemiluminescent signal was backfitted onto a standard curve using a four-parameter fit within the GraphPad Prism software package.

### Purification of LMIV230–01 scFv and formation of the antigen–scFv complex

The variable segment of the heavy chain sequence of LMIV230-01 was fused to the light chain variable segment by a (GGGGS)_5_ linker and cloned into the vector pHLsec^46^. This plasmid was transfected into Expi293 cells with ExpiFectamine (Thermo Fisher Scientific, Waltham, USA) according to the manufacturer’s protocol. Recombinant LMIV230–01 scFv expressed as secreted protein was harvested on day 5 post-transfection. The culture medium was loaded into a Ni Sepharose excel (GE Healthcare, Pittsburgh, USA) column and the resin was washed with Buffer A (25 mM Tris pH 7.4, 0.3 M NaCl) supplemented with 30 mM imidazole. LMIV230–01 scFv was eluted with Buffer A supplemented with 150 mM imidazole. Eluate was concentrated and injected onto a Superdex75 column (GE Healthcare, Pittsburgh, USA) equilibrated with 20 mM Tris. pH 8, 100 mM NaCl. Fractions were pooled and stored at −80 °C. Purified LMIV230–01 scFv protein was mixed with Pfs230D1 in 1:1 molar ratio on ice for 30 min to allow complex formation. The complex was concentrated and injected onto a Superdex75 column equilibrated with 20 mM Tris. pH 8, 100 mM NaCl. Fractions of the complex were pooled and stored at −80 °C.

### Crystallization, data collection, and structure determination of the Pfs230D1–LMIV230–01 scFv complex

Crystals of Pfs230D1–LMIV230–01 scFv were grown using sitting-drop vapor diffusion at 18 °C. The protein solution (10 mg/ml) was mixed with 0.1 M MES, pH 6.5, 26% PEG 3350 in a 1:1 ratio for crystallization. The crystals were cryoprotected in well solution supplemented with 20% glycerol prior to flash freezing in liquid nitrogen. X-ray diffraction experiments were carried out on the GM/CA beamlines, Advanced Photon Source (APS), Argonne National Laboratory at 100 K. Diffraction data were processed with XDS^47^. The structure of the Pfs230D1–LMIV230–01 scFv complex was determined by molecular replacement in PHASER^48^ using a model of LMIV230–01 scFv created by the PIGS server^49^. Iterative model building using AutoBuild in PHENIX^50^ and COOT^51^, and refinement using Refmac^52^ led to the current model for Pfs230D1–LMIV230–01 scFv (*R*_work_*/R*_free_ of 0.18/0.23). Data collection and refinement statistics are shown in Supplementary Table 3. Epitopes were identified by determining interfaces between the scFv and antigen using PDBePISA^53^.

### Statistics and reproducibility

All experiments were independently reproduced three times unless stated otherwise in the figure legend.

### Reporting summary

Further information on research design is available in the Nature Research Reporting Summary linked to this article.

## Supplementary information

Supplementary Information

Reporting Summary

## Data Availability

All data generated or analyzed during this study are included in this published article (and its Supplementary Information files). Sequences of LMIV230-01 and LMIV230-02 are available in Supplementary Table 7. Atomic coordinates and structural factors of Pfs230D1–LMIV230-01 scFv were deposited to the Protein Data Bank under accession code 7JUM. Source data are provided with this paper.

## Source data

Source Data

## References

[1] Eksi S (2006). Malaria transmission-blocking antigen, Pfs230, mediates human red blood cell binding to exflagellating male parasites and oocyst production. Mol. Microbiol.

[2] Williamson KC, Keister DB, Muratova O, Kaslow DC (1995). Recombinant Pfs230, a Plasmodium falciparum gametocyte protein, induces antisera that reduce the infectivity of Plasmodium falciparum to mosquitoes. Mol. Biochem. Parasitol..

[3] Tachibana M (2019). Identification of domains within Pfs230 that elicit transmission blocking antibody responses. Vaccine.

[4] Coelho CH, Rappuoli R, Hotez PJ, Duffy PE (2019). Transmission-blocking vaccines for malaria: time to talk about vaccine introduction. Trends Parasitol..

[5] Coelho, C. H. et al. Antimalarial antibody repertoire defined by plasma IG proteomics and single B cell IG sequencing. *JCI Insight* **5**, e143471 (2020). 10.1172/jci.insight.143471 PMC7710313 33048842

[6] Singh K (2020). Structure and function of a malaria transmission blocking vaccine targeting Pfs230 and Pfs230-Pfs48/45 proteins. Commun. Biol..

[7] Kisalu NK (2018). A human monoclonal antibody prevents malaria infection by targeting a new site of vulnerability on the parasite. Nat. Med..

[8] Alanine DGW (2019). Human antibodies that slow erythrocyte invasion potentiate malaria-neutralizing antibodies. Cell.

[9] Rappuoli R, Bottomley MJ, D’Oro U, Finco O, De Gregorio E (2016). Reverse vaccinology 2.0: Human immunology instructs vaccine antigen design. J. Exp. Med..

[10] Triller G (2017). Natural parasite exposure induces protective human anti-malarial antibodies. Immunity.

[11] Tan J (2016). A LAIR1 insertion generates broadly reactive antibodies against malaria variant antigens. Nature.

[12] Watson CT (2013). Complete haplotype sequence of the human immunoglobulin heavy-chain variable, diversity, and joining genes and characterization of allelic and copy-number variation. Am. J. Hum. Genet..

[13] Avnir Y (2014). Molecular signatures of hemagglutinin stem-directed heterosubtypic human neutralizing antibodies against influenza A viruses. PLoS Pathog..

[14] Avnir Y (2016). IGHV1-69 polymorphism modulates anti-influenza antibody repertoires, correlates with IGHV utilization shifts and varies by ethnicity. Sci. Rep..

[15] Lensen A (1996). Measurement by membrane feeding of reduction in Plasmodium falciparum transmission induced by endemic sera. Trans. R. Soc. Trop. Med Hyg..

[16] Miura K (2016). Transmission-blocking activity is determined by transmission-reducing activity and number of control oocysts in Plasmodium falciparum standard membrane-feeding assay. Vaccine.

[17] Doolan KM, Colby DW (2015). Conformation-dependent epitopes recognized by prion protein antibodies probed using mutational scanning and deep sequencing. J. Mol. Biol..

[18] Verrier FC (1997). Antibodies to several conformation-dependent epitopes of gp120/gp41 inhibit CCR-5-dependent cell-to-cell fusion mediated by the native envelope glycoprotein of a primary macrophage-tropic HIV-1 isolate. Proc. Natl Acad. Sci. USA.

[19] Outchkourov N (2007). Epitope analysis of the malaria surface antigen pfs48/45 identifies a subdomain that elicits transmission blocking antibodies. J. Biol. Chem..

[20] Healer J (1997). Complement-mediated lysis of Plasmodium falciparum gametes by malaria-immune human sera is associated with antibodies to the gamete surface antigen Pfs230. Infect. Immun..

[21] Tegla CA (2011). Membrane attack by complement: the assembly and biology of terminal complement complexes. Immunol. Res..

[22] Bayly-Jones, C., Bubeck, D. & Dunstone, M. A. The mystery behind membrane insertion: a review of the complement membrane attack complex. *Philos. Trans. R. Soc. Lond. B: Biol. Sci.* **372** (2017). 10.1098/rstb.2016.0221 PMC5483522 28630159

[23] Holm L, Rosenstrom P (2010). Dali server: conservation mapping in 3D. Nucleic Acids Res..

[24] Doritchamou JYA (2019). Placental malaria vaccine candidate antigen VAR2CSA displays atypical domain architecture in some Plasmodium falciparum strains. Commun. Biol..

[25] Collins WE, Warren M, Skinner JC, Richardson BB, Kearse TS (1977). Infectivity of the Santa Lucia (El Salvador) strain of Plasmodium falciparum to different anophelines. J. Parasitol..

[26] MacDonald NJ (2016). Structural and immunological characterization of recombinant 6-cysteine domains of the plasmodium falciparum sexual stage protein Pfs230. J. Biol. Chem..

[27] Farrance CE (2011). A plant-produced Pfs230 vaccine candidate blocks transmission of Plasmodium falciparum. Clin. Vaccin. Immunol..

[28] Singh SK (2019). Pfs230 and Pfs48/45 fusion proteins elicit strong transmission-blocking antibody responses against Plasmodium falciparum. Front Immunol..

[29] Chen E, Salinas ND, Ntumngia FB, Adams JH, Tolia NH (2015). Structural analysis of the synthetic Duffy Binding Protein (DBP) antigen DEKnull relevant for Plasmodium vivax malaria vaccine design. PLoS Negl. Trop. Dis..

[30] Dormitzer PR, Grandi G, Rappuoli R (2012). Structural vaccinology starts to deliver. Nat. Rev. Microbiol.

[31] Healy, S. A. et al. Pfs230 yields higher malaria transmission-blocking vaccine activity than Pfs25 in humans but not mice. *J. Clin. Invest.*10.1172/JCI146221 (2021). 10.1172/JCI146221 PMC8011888 33561016

[32] Talaat KR (2016). Safety and immunogenicity of Pfs25-EPA/Alhydrogel(R), a transmission blocking vaccine against Plasmodium falciparum: an open label study in malaria naive adults. PLoS ONE.

[33] Krishnamurty AT (2016). Somatically hypermutated plasmodium-specific IgM(+) memory B cells are rapid, plastic, early responders upon malaria rechallenge. Immunity.

[34] Taylor JJ (2012). Deletion and anergy of polyclonal B cells specific for ubiquitous membrane-bound self-antigen. J. Exp. Med..

[35] Yang Y (2015). Distinct mechanisms define murine B cell lineage immunoglobulin heavy chain (IgH) repertoires. Elife.

[36] Lefranc MP (1999). IMGT, the international ImMunoGeneTics database. Nucleic Acids Res..

[37] Alamyar E, Duroux P, Lefranc MP, Giudicelli V (2012). IMGT((R)) tools for the nucleotide analysis of immunoglobulin (IG) and T cell receptor (TR) V-(D)-J repertoires, polymorphisms, and IG mutations: IMGT/V-QUEST and IMGT/HighV-QUEST for NGS. Methods Mol. Biol..

[38] Galson JD (2015). In-depth assessment of within-individual and inter-individual variation in the b cell receptor repertoire. Front Immunol..

[39] Gupta NT (2015). Change-O: a toolkit for analyzing large-scale B cell immunoglobulin repertoire sequencing data. Bioinformatics.

[40] Stern JN (2014). B cells populating the multiple sclerosis brain mature in the draining cervical lymph nodes. Sci. Transl. Med..

[41] Bodenhofer U, Bonatesta E, Horejs-Kainrath C, Hochreiter S (2015). msa: an R package for multiple sequence alignment. Bioinformatics.

[42] Wickham, H. *ggplot2: Elegant Graphics for Data Analysis* (Springer, 2016).

[43] Han A, Glanville J, Hansmann L, Davis MM (2014). Linking T-cell receptor sequence to functional phenotype at the single-cell level. Nat. Biotechnol..

[44] Coelho CH (2019). Chronic helminth infection does not impair immune response to malaria transmission blocking vaccine Pfs230D1-EPA/Alhydrogel(R) in mice. Vaccine.

[45] Wu Y (2008). Phase 1 trial of malaria transmission blocking vaccine candidates Pfs25 and Pvs25 formulated with montanide ISA 51. PLoS ONE.

[46] Aricescu AR, Lu W, Jones EY (2006). A time- and cost-efficient system for high-level protein production in mammalian cells. Acta Crystallogr. D: Biol. Crystallogr..

[47] Kabsch WXds (2010). Acta Crystallogr. D: Biol. Crystallogr..

[48] McCoy AJ (2007). Phaser crystallographic software. J. Appl. Crystallogr..

[49] Marcatili P, Rosi A, Tramontano A (2008). PIGS: automatic prediction of antibody structures. Bioinformatics.

[50] Adams PD (2002). PHENIX: building new software for automated crystallographic structure determination. Acta Crystallogr. D: Biol. Crystallogr..

[51] Emsley P, Cowtan K (2004). Coot: model-building tools for molecular graphics. Acta Crystallogr. D: Biol. Crystallogr..

[52] Collaborative Computational Project, N. (1994). The CCP4 suite: programs for protein crystallography. Acta Crystallogr. D: Biol. Crystallogr..

[53] Krissinel E, Henrick K (2007). Inference of macromolecular assemblies from crystalline state. J. Mol. Biol..

