# Author Correction: A human monoclonal antibody blocks malaria transmission and defines a highly conserved neutralizing epitope on gametes

**DOI:** 10.1038/s41467-022-32574-9

**Published:** 2022-08-15

**Authors:** Camila H. Coelho, Wai Kwan Tang, Martin Burkhardt, Jacob D. Galson, Olga Muratova, Nichole D. Salinas, Thiago Luiz Alves e Silva, Karine Reiter, Nicholas J. MacDonald, Vu Nguyen, Raul Herrera, Richard Shimp, David L. Narum, Miranda Byrne-Steele, Wenjing Pan, Xiaohong Hou, Brittany Brown, Mary Eisenhower, Jian Han, Bethany J. Jenkins, Justin Y. A. Doritchamou, Margery G. Smelkinson, Joel Vega-Rodríguez, Johannes Trück, Justin J. Taylor, Issaka Sagara, Sara A. Healy, Jonathan P. Renn, Niraj H. Tolia, Patrick E. Duffy

**Affiliations:** 1 Pathogenesis and Immunity Section, Laboratory of Malaria Immunology and Vaccinology, National Institute of Allergy and Infectious Diseases, National Institutes of Health, Bethesda, MD USA; 2 Host-Pathogen Interactions and Structural Vaccinology Section, Laboratory of Malaria Immunology and Vaccinology, National Institute of Allergy and Infectious Diseases, National Institutes of Health, Bethesda, MD USA; 3 Vaccine Development Unit, Laboratory of Malaria Immunology and Vaccinology, National Institute of Allergy and Infectious Diseases, National Institutes of Health, Bethesda, MD USA; 4 Division of Immunology and Children’s Research Center, University Children’s Hospital Zurich, University of Zurich (UZH), Zurich, Switzerland; 5 Alchemab Therapeutics Ltd, 55-56 Russell Square, London, UK; 6 Laboratory of Malaria and Vector Research, National Institute of Allergy and Infectious Diseases, National Institutes of Health, Rockville, MD USA; 7 iRepertoire Inc., Huntsville, AL USA; 8 Biological Imaging Section, National Institute of Allergy and Infectious Diseases, National Institutes of Health, Bethesda, MD USA; 9 Fred Hutchinson Cancer Research Center, Seattle, WA USA; 10 Malaria Research and Training Center, University of Sciences, Techniques, and Technology, Bamako, Mali

**Keywords:** Protein vaccines, Parasite host response

Correction to: *Nature Communications* 10.1038/s41467-021-21955-1, published online 19 March 2021.

The original version of this Article omitted from the author list the 27th author Sara A. Healy, who is from the ‘Vaccine Development Unit, Laboratory of Malaria Immunology and Vaccinology, National Institute of Allergy and Infectious Diseases, National Institutes of Health, Bethesda, MD, USA’. Consequently, ‘S.A.H.’ was added to the Author Contributions: ‘I.S., S.A.H., J.J.T., J.V.R., J.T., J.H., M.B.S., J.R., N.H.T., and P.E.D. supervised the experiments and interpreted the data.’ This has been corrected in both the PDF and HTML versions of the Article.

