# Supplementary material for: A human monoclonal antibody blocks malaria transmission and defines a highly conserved neutralizing epitope on gametes
Source: Nat Commun. 2021 Mar 19;12:1750. doi: 10.1038/s41467-021-21955-1 (PMC7979743; doi:10.1038/s41467-021-21955-1)
Supplement: Supplementary file 1 — Supplementary Information [file 41467_2021_21955_MOESM1_ESM.pdf]

SUPPLEMENTARY MATERIAL (FIGURES AND TABLES)

FIGURES

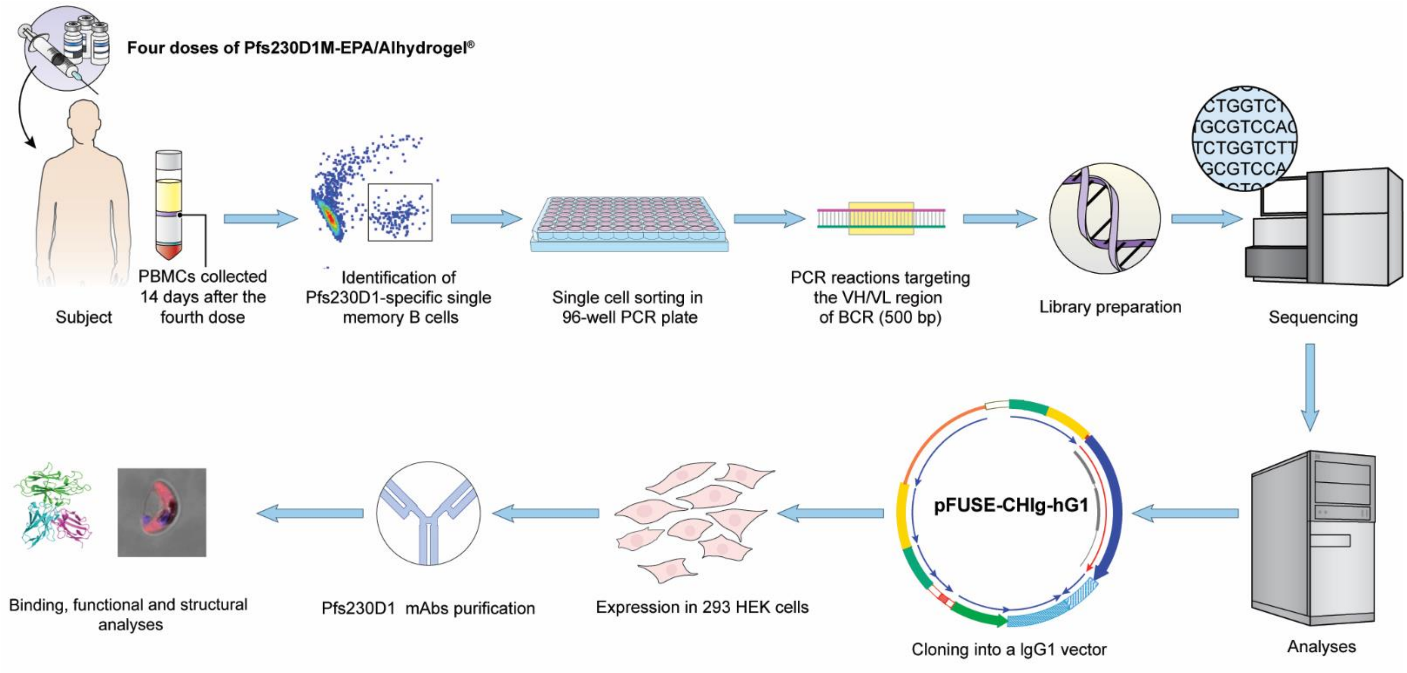

Supplementary Fig. 1| Experimental pipeline. Pfs230D1-specific single B cells were sorted from PBMCs of eight Malian adults who had been immunized with four doses of 40µg of Pfs230D1-EPA/Alhydrogel®. After extraction of single B cells, a 500 bp fragment of the BCR variable regions of VH/VL were amplified and sequenced. Matched VH/VL pairs that were identified in more than one B cell were preferentially selected for cloning in an IgG1 vector for expression in 293 HEK cells and subsequent analyses.

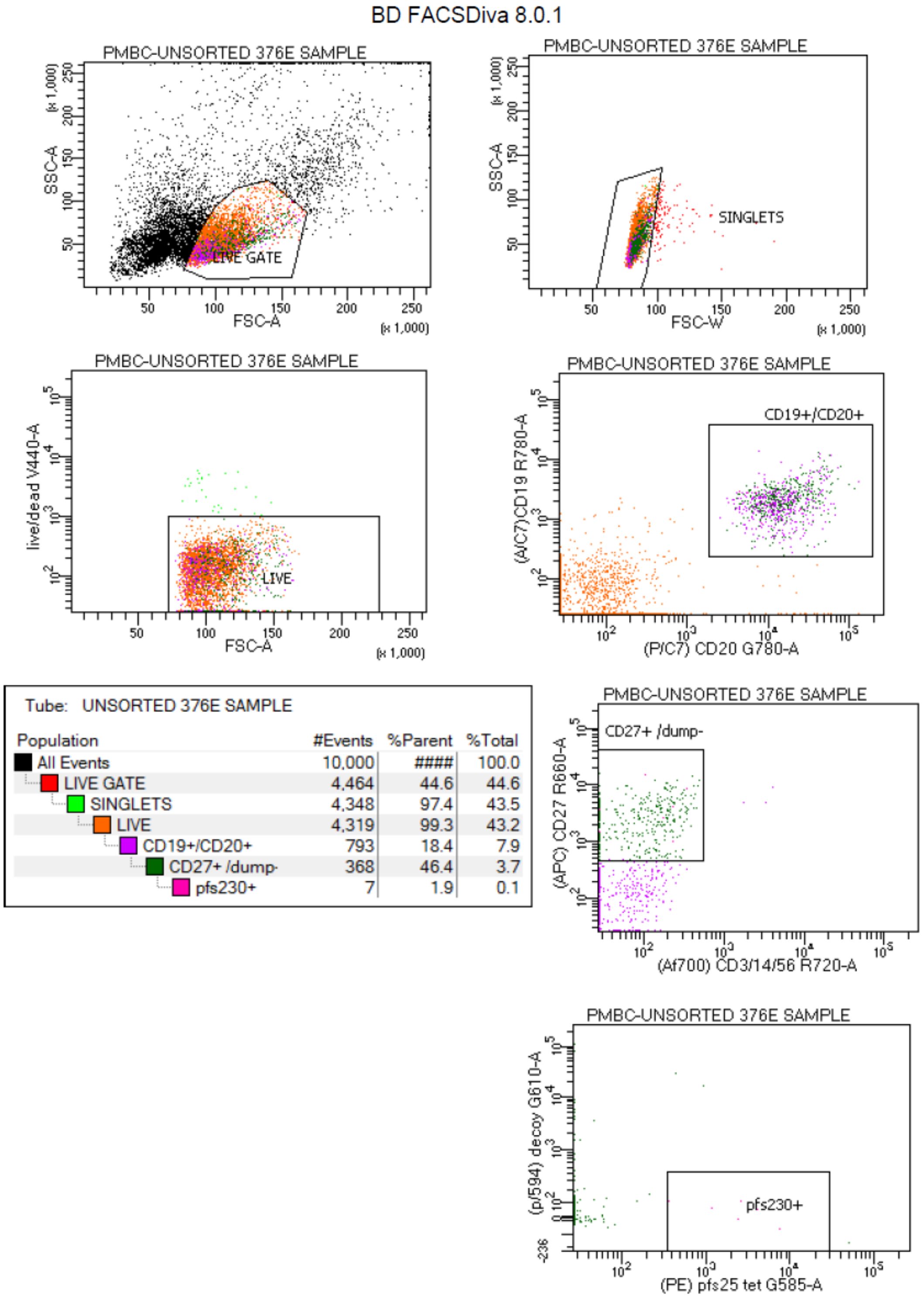

Population	#Events	%Parent	%Total
All Events	10,000	####	100.0
LIVE GATE	4,464	44.6	44.6
SINGLETS	4,348	97.4	43.5
LIVE	4,319	99.3	43.2
CD19+/CD20+	793	18.4	7.9
CD27+ /dump-	368	46.4	3.7
pfs230+	7	1.9	0.1

Supplementary Fig. 2| Flow cytometry gating strategy to sort Pfs230D1-specific B cells from PBMCs collected after 4 doses of Pfs230D1-EPA/Alhydrogel.

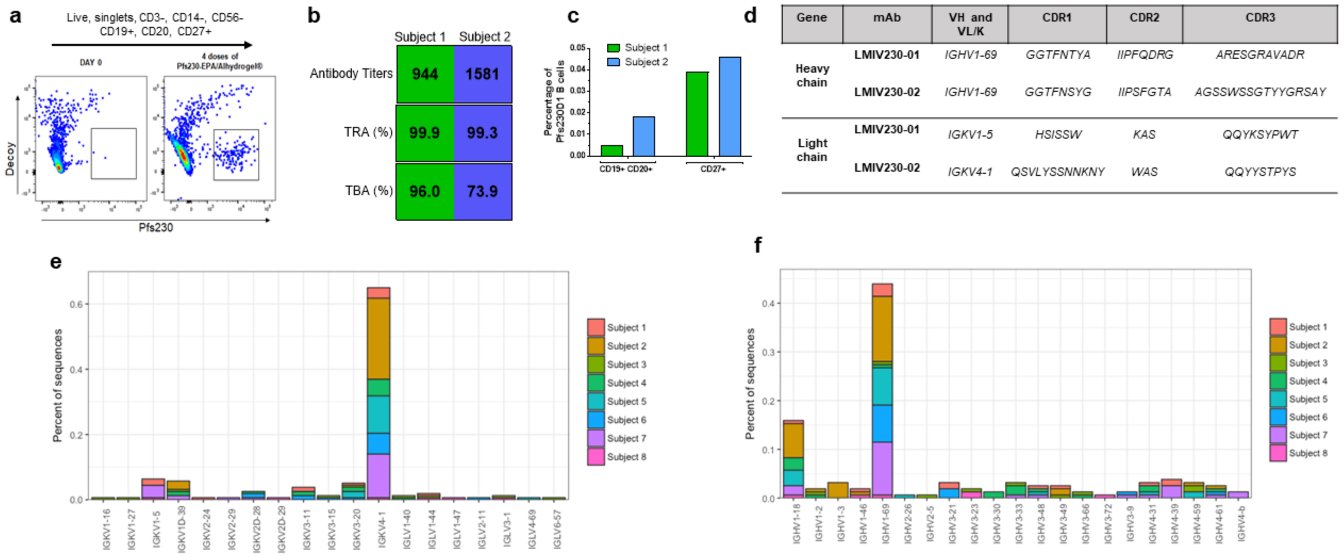

Supplementary Fig. 3| Pfs230D1-specific mAbs belong to the same heavy chain germline subgroup but differ for kappa chain. a, Sorted memory B cells were gated as live, single cells, excluded for CD3, CD14 and CD56, and gated on CD19+, CD20+, CD27+ cells. Then, a tetramer approach was used to select antigen-specific cells and reduce nonspecific binding. Cells binding to the decoy tetramer (BSA) were excluded and only those binding to Pfs230D1 were selected for sorting. **b,** Serum from each subject was used to measured antibody titers against Pfs230D1 and functional activity to reduce oocyst burden in Standard Membrane Feeding Assays (SMFA). TRA= Transmission Reducing Activity measured as the reduction in average oocyst count; TBA= Transmission Blocking Activity measured as the reduction in the proportion of infected mosquitoes. **c,** Proportion of memory B cells for each subject that are Pfs230D1-specific. Only one vial of PBMC was available, and this analysis was performed just once **d,** Complementarity-determining regions (CDRs) of each sequence selected for mAb expression. **e,** IGKV4-1 germline (gene sequence in LMIV230-02) was the most frequent for the kappa chain genes. IGKV1-5 germline (gene sequence in LMIV230-01) was found in only three subjects **f,** Sequences related to germline 1-69 of the IGHV gene were the most frequently elicited in response to the vaccination. Source data are provided as a Source Data file.

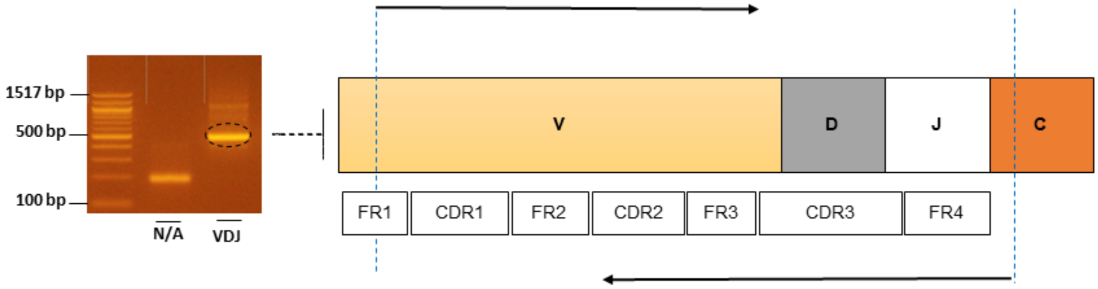

Supplementary Fig. 4| Amplification of V(D)J region. 500 bp fragment amplified from cDNA of sorted Pfs230D1-specific single B cell. This fragment was obtained using primers targeting the V(D)J region (iRepertoire Inc.).

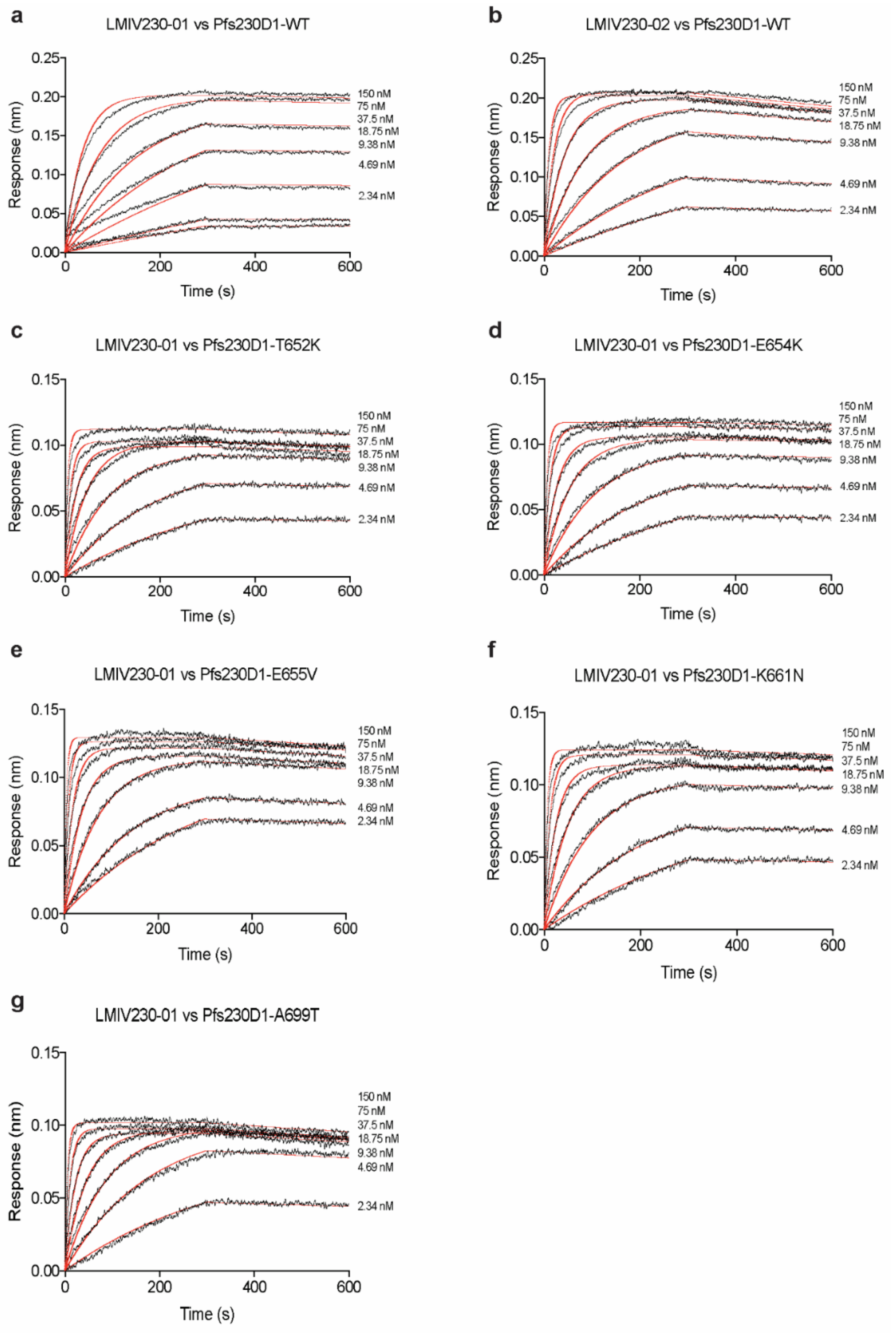

Supplementary Fig 5| Affinity measurements of anti-Pfs230D1 mAbs to recombinant Pfs230D1 variants. a-b, Representative sensorgrams of LMIV230-01 and LMIV230-02 to the recombinant Pfs230D1 wild type protein. **c-g,** Representative sensorgrams of LMIV230-1 to Pfs230D1 polymorphisms. Two biological replicates with three technical replicates each were performed. All sensorgrams were globally fitted with 1:1 binding interaction.

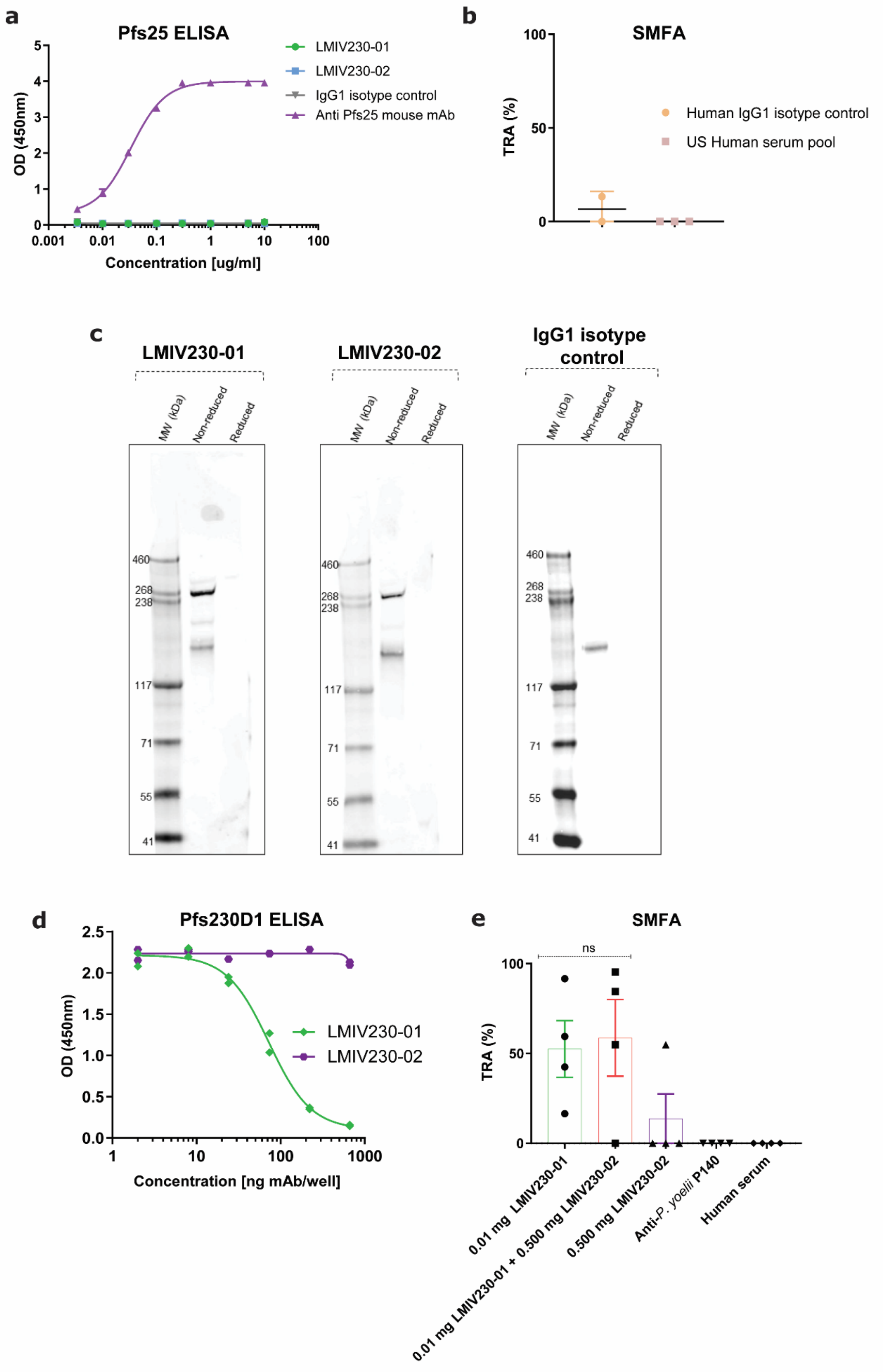
a
Pfs25 ELISA
OD (450nm)
Concentration [ug/ml]
LMIV230-01
LMIV230-02
IgG1 isotype control
Anti Pfs25 mouse mAb
b
SMFA
TRA (%)
Human IgG1 isotype control
US Human serum pool
c
LMIV230-01
LMIV230-02
IgG1 isotype control
MW (kDa)
Non-reduced
Reduced
460
268
238
117
71
55
41
d
Pfs230D1 ELISA
OD (450nm)
Concentration [ng mAb/well]
LMIV230-01
LMIV230-02
e
SMFA
TRA (%)
ns
0.01 mg LMIV230-01
0.01 mg LMIV230-01 + 0.500 mg LMIV230-02
0.500 mg LMIV230-02
Anti-P. yoelii P140
Human serum

Supplementary Fig.6| Additional binding and functional characterization of LMIV230-01 and -02. a, Both mAbs failed to bind to the ookinete protein Pfs25. **b,** Additional controls for the Standard Membrane Feeding Assay (SMFA). Human IgG1 isotype control was expressed using the same conditions as LMIV230-01 and -02 and was used in this assay at 1000µg/mL. Sixty microliters of undiluted human pooled serum obtained from US healthy donors were used as additional control. Values are shown as mean ± s.e.m. **c,** Full depiction of the Western blot gel displayed in Fig. 1g .**d,** The two mAbs do not compete for the same epitope in the recombinant Pfs230D1 protein, since unlabelled LMIV230-01 blocks binding of LMIV-230-01-HRP to immobilized Pfs230D1 but LMIV230-02 does not. **e,** Combination of LMIV230-01 and LMIV230-02 did not increase functional activity over LMIV230-01 alone. Control mosquitoes were fed with mouse IgG1 mAb targeting *P. yoelii* P140 protein, or with non-immune human serum. Statistics were performed using One-Way ANOVA followed by Tukey's multiple comparisons. Bars represent mean and SEM. Source data are provided as a Source Data file.

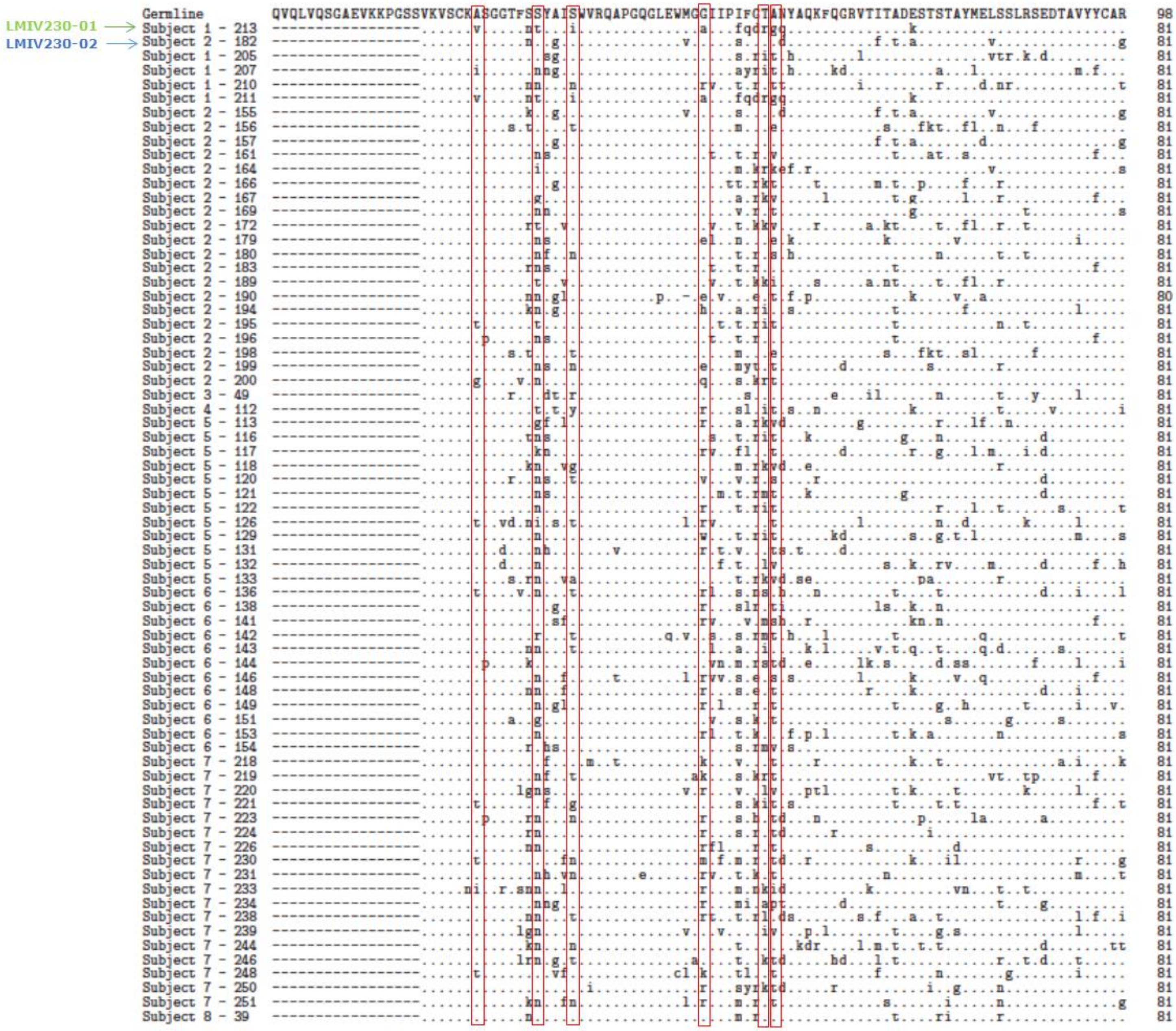

Supplementary Fig.7| Mutations compared to IGHV1-69 germline sequence. Figure shows CDR1 and CDR2 sequences from Pfs230D1-single memory B cells using IGHV1-69 V segment**.** Sequences from 8 individuals vaccinated with four doses Pfs230-EPA/Alhydrogel® are compared to the IGHV1-69*01 germline from IMGT®. Any lower-case letter is a mutation, and there are approximately 10 aa mutations per sequence, suggesting they have undergone proliferation and selection in response to the vaccine. Specific changes (highlighted in red) at positions 24, 31, 35, 50, 57 and 58 are shared among most of sequences, and this could indicate that novel mutations in these positions have some functional importance for the antibodies, or alternatively that it is a novel allele.

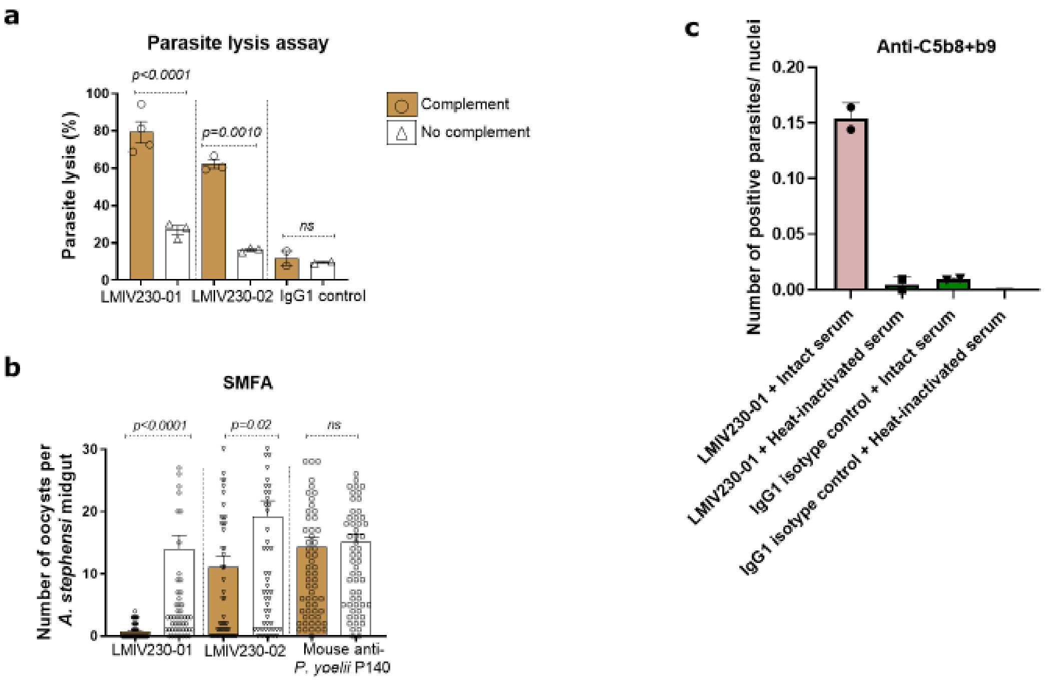

Supplementary Fig.8| Pfs230 mAbs activity is complement-dependent and LMIV230-01 competing antibodies are acquired at varying levels by vaccinees. a, Activity of LMIV230-01 and LMIV230-02 is complement-dependent in the vitro lysis assay and **b,** in the vivo mosquito feeding assay. **c,** Membrane attack complexes (MAC) on parasites were detected using an Alexa 488-labeled antibody that recognizes the assembled MAC complex (anti C5b-9+ C5b-8). Gametes incubated with LMIV230-01 and intact serum produced MAC-positive parasites. Heat-inactivating serum to degrade the heat-labile components of the complement pathway eliminated deposition of MAC on gametes. MAC-positive *P. falciparum* strain NF54 gametes were enumerated in a large, tiled confocal image and normalized to the number of Hoechst-stained nuclei. One-Way ANOVA followed by Tukey’s multiple comparisons was used for a-c. Bars represent mean and SEM. Source data are provided as a Source Data file.

a

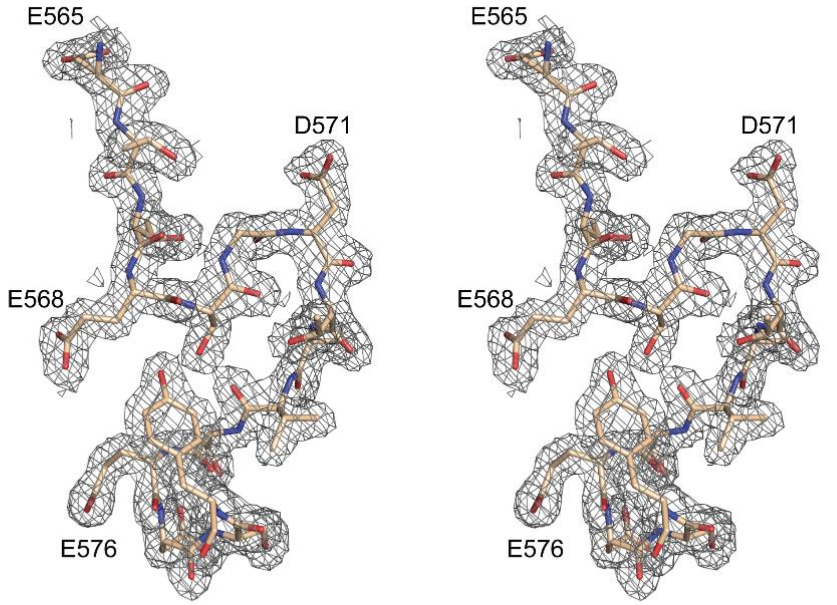

b

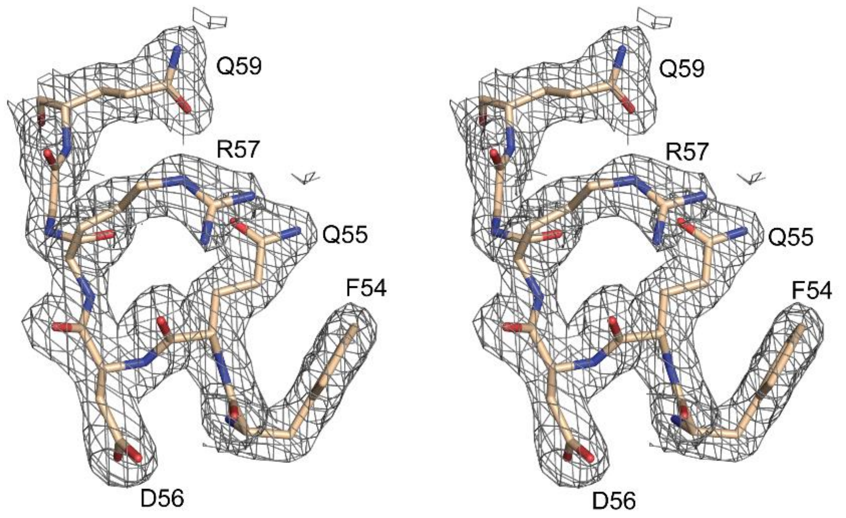

Supplementary Fig. 9| Stereo view of representative electron density of the Pfs230D1-LMIV230-01scFv complex. a, *2Fo-Fc* map (gray mesh) of the N-terminal loop of Pfs230D1 (stick model) that interacts with LMIV230-01scFv at 1.0 σ level. **b,** *2Fo-Fc* map (gray mesh) of the CDR3 region of LMIV230-01scFv heavy chain (stick model) at 1.0 σ level.

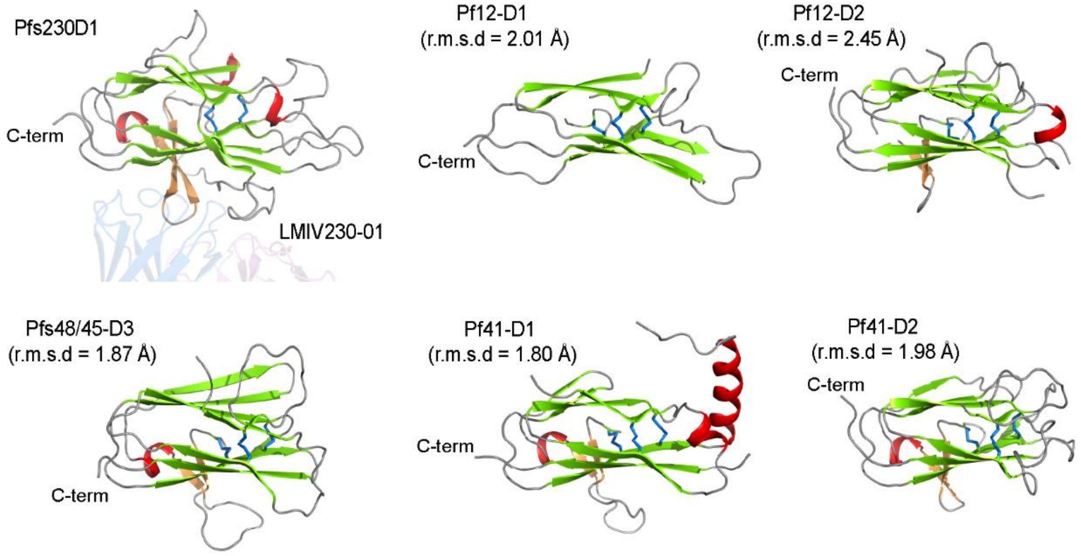

Supplementary Fig. 10| Homology structures of Pfs230D1. Secondary structure cartoon representation of Pfs230D1 in comparison to known 6-Cys domains of highest homology (Pf12, PDB:2YMO[53]; Pf41, PDB:4YS4[54]; Pfs48/45, PDB:6E62[55]). Beta-sandwiches in green, beta strands in orange, helices in red, loops in gray and disulfide bonds in blue sticks. LMIV230-01scFv is semi-transparent to show the binding epitope on Pfs230D1. The C-terminus of each structure is labelled. Root mean square deviations (r.m.s.d.) between Pfs230D1 (176 residues) and individual 6-Cys domains are shown.

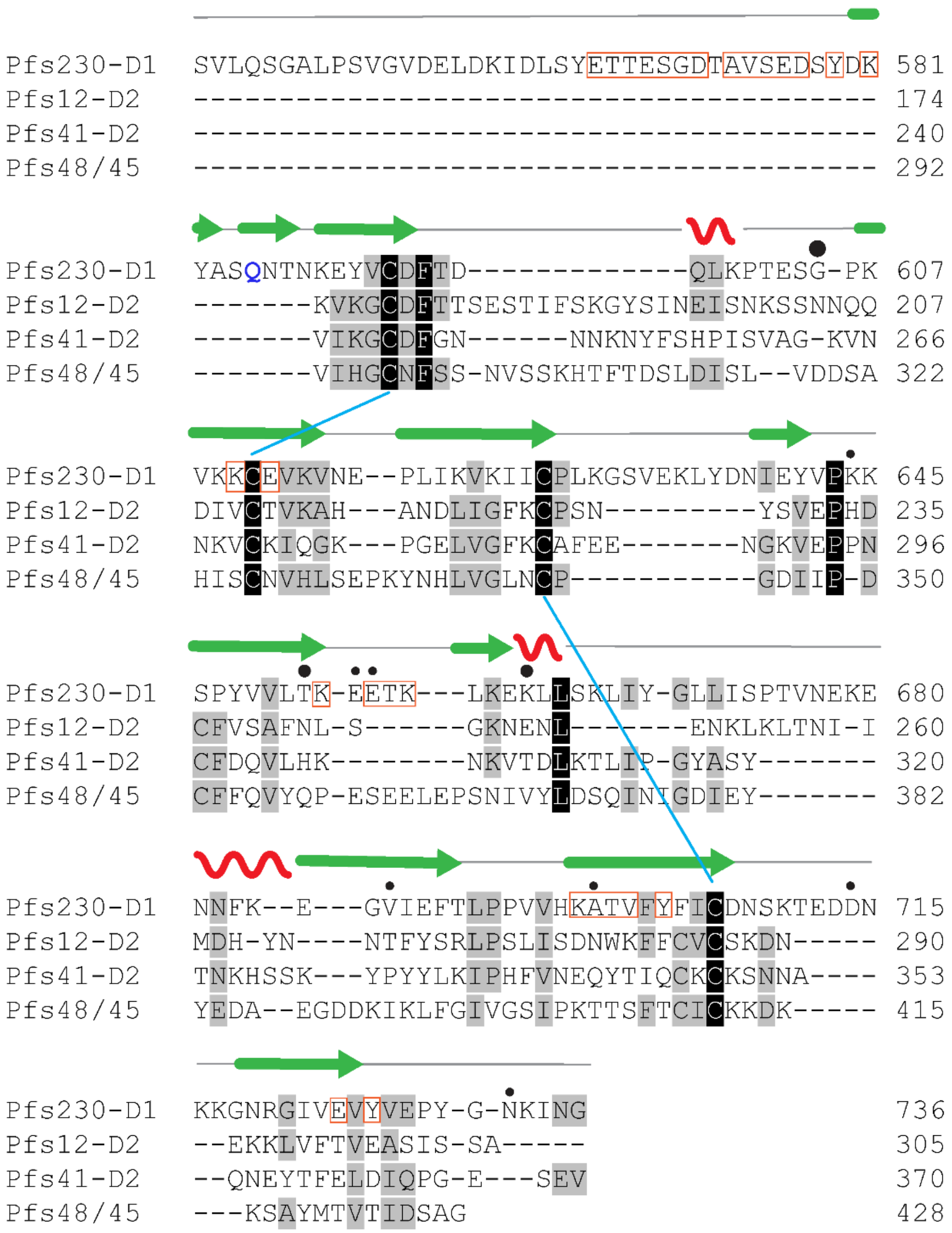

Supplementary Fig. 11| Secondary structure sequence alignment of Pfs230D1 with proteins of highest structural similarity calculated by DALI server[23]. Secondary structure elements of Pfs230D1 are shown with arrows and ribbons representing strands and helices, respectively. Disulfide bonds are linked with blue lines. Residues contacted by LMIV230-01scFv are in orange boxes. Mutation site (N585Q) to prevent N-glycosylation in *P. pastoris* is bolded in blue. Black circles above the sequence indicate polymorphisms. Bigger circles represent higher frequency.

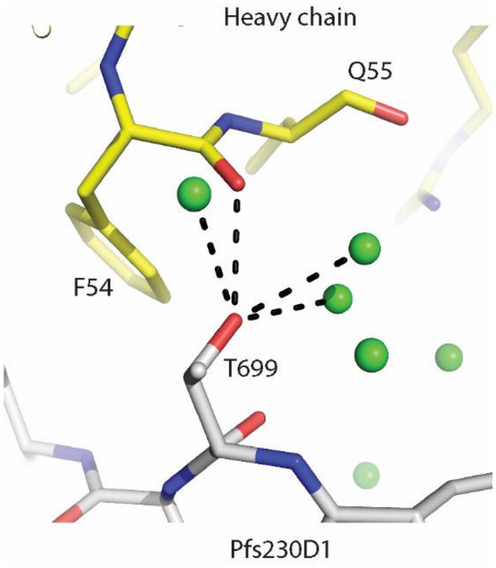

Supplementary Fig. 12| Model of A699T mutant of Pfs230D1. Amino acids are represented in stick models. Carbon in yellow (heavy chain of LMIV230-01)/grey (Pfs230D1); nitrogen in blue; oxygen in red. Water molecules are in green spheres. Potential interactions are drawn as dashed lines.

a

```
                                 CDR1                      CDR2                    CDR3
                  20        30        40     50      60            100       110        120
LMIV230-01_heavy  KVSCKVSGGTF....NTYAII.//..LEWMGAIIPF..QDRGQY..//.AVYYCAKESGRAVADRWGQGTLVTVSS
IGHV1-69*04F (V)  KVSCKASGGTF....SSYAIS.//..LEWMGGIIPI..LGIANY..//.AVYYCAR
IGHD6-19*01F (D)                                                          ESGRAVA
IGHJ5*02F (J)                                                                    DPWGQGTLVTVSS
```

b

```
                             CDR1                      CDR2                      CDR3
                     30          40          50        60                    110        120
LMIV230-01_light  CRASHSI......SSWLAW..//..GKAPKLLIYKA.......STL...//..YYCQQYKSYPWTFGQGTKVEIK
IGKV1-5*03F (V)   CRASQSI......SSWLAW..//..GKAPKLLIYKA.......SSL...//..YYCQQYNS
IGKJ*01F (J)                                                                   YPWTFGQGTKVEIK
```

c

```
                                                  CDR1                 CDR2
LMIV230-01 heavy      QVQLVQSGAEVKKPGSSVKVSCKVSGGTFNTYAIIWVRQAPGQGLEWMGAIIPFQDRGQY     60
LMIV230-02 heavy      QVQLVQSGAEVKKPGSSVKVSCKASGGTFNSYGISWVRQAPGQGLEWVGGIIPSFGTADY     60
                                             *      * * *            * *   ******

                                                                    CDR3
LMIV230-01 heavy      AQKFQGRVTITADKSTSTAYMELSSLRSEDTAVYYCAKESGR-----AVADRWGQGTLVTVSS 118
LMIV230-02 heavy      AQKFQGRVTFTTDTSTSTAYMEVSSLRSEDTAVYYCAGSSWSSGTYYGRSAYWGQGTLVTVSS 123
                               * * *        *              ** ************
```

d

```
                                                            CDR1                    CDR2
LMIV230-01 light      DIVMTQSPASLALSLGERATLYCRASHSI------SSWLAWYQQKPGKAPKLLIYKASTL     54
LMIV230-02 light      EIVMTQSPASLALSLGERATINCKSSQSVLYSSNNKNYLAWYQQKPGQPPKLLIYWASTR     60
                      *                   ** ** ************         *       *

                                                                   CDR3
LMIV230-01 light      ESGVPSRFSGSGSGTEFTLTISSLQPDDFATYYCQQYKSYPWTFGQGTKLEIKR 108
LMIV230-02 light      ESGVPDRFSGSGSGTDFTLTISSLQAGDVAVYHCQQYYSTPYSFGQGTKLEIKR 114
                           *         *         ** * * *    * * **
```

Supplementary Fig. 13| Monoclonal antibody maturation. a,b, Amino acid sequence alignment of the variable region of (a) heavy and (b) light chains of LMIV230-01 against the germline V,D and J gene members with the highest sequence similarity, defined by NCBI IgBlast and IMGT/V-Quest as indicated in each alignment. CDRs are shown at the top of the alignment. Amino acid mutations are highlighted in cyan. **c,d,** Amino acid sequence alignment of the (c) V-D-J-region of heavy and (d) V-J-region of light chain of LMIV230-01 and LMIV230-02 mAbs. CDRs are shown at the top of the alignment. Non-identical residues are labelled with asterisks.

```
NF54          SVLQSGALPSVGVDELDKIDLSYETTESGDTAVSEDSYDKYASNNTNKEYVCDFTDQLKP    601
20-2217-0     SVLQSGALPSVGVDELDKIDLSYETTESGDTAVSEDSYDKYASNNTNKEYVCDFTDQLKP    601
St.Lucia      SVLQSGALPSVGVDELDKIDLSYETTESGDTAVSEDSYDKYASNNTNKEYVCDFTDQLKP    601
              ************************************************************

NF54          TESGPKVKKCEVKVNEPLIKVKIICPLKGSVEKLYDNIEYVPKKSPYVVLTKEETKLKEK    661
20-2217-0     TESSPKVKKCEVKVNEPLIKVKIICPLKGSVEKLYDNIEYVPKKSPYVVLTKEETKLKEK    661
St.Lucia      TESSPKVKKCEVKVNEPLIKVKIICPLKGSVEKLYDNIEYVPKKSPYVVLTKEETKLKEN    661
              ***.*******************************************************:

NF54          LLSKLIYGLLISPTVNEKENNFKEGVIEFTLPPVVHKATVFYFICDNSKTEDDNKKGNRG    721
20-2217-0     LLSKLIYGLLISPTVNEKENNFKEGVIEFTLPPVVHKATVFYFICDNSKTEDDNKKGNRG    721
St.Lucia      LLSKLIYGLLISPTVNEKENNFKEGVIEFTLPPVVHKATVFYFICDNSKTEDDNKKGNRG    721
              ************************************************************

NF54          IVEVYVEPYGNKING   736
20-2217-0     IVEVYVEPYGNKING   736
St.Lucia      IVEVYVEPYGNKING   736
              ***************
```

Supplementary Fig. 14| Pfs230D1 polymorphisms in the other *P. falciparum* strains analyzed. DNA sequencing revealed that the Malian isolate 20-2217-0 and St. Lucia strain contain the G605S/R polymorphism while St. Lucia also contains the K661N polymorphism; both polymorphisms reside outside the LMIV230-01 binding epitope.

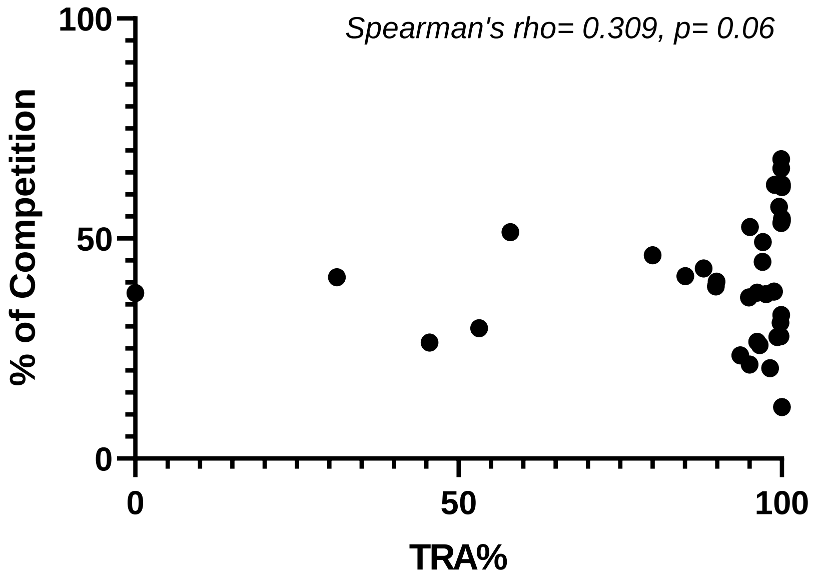

Supplementary Fig. 15| Correlation between levels of LMIV230-01 competing antibodies and Transmission-Reducing Activity (TRA) measured in SMFA. Source data are provided as a Source Data file.

SUPPLEMENTARY MATERIAL

<u>TABLES</u>

**Subject ID**	**Antibody titers**	**TRA (%)**	**TBA (%)**
1	944	99.9	96
2	1581	99.3	73.9
3	2115	100	100
4	1382	99.6	79.2
5	2100	100	100
6	5277	100	100
7	800	99.9	95.8
8	774	95.1	20.8

Supplementary Table 1| Antibody titers and functional activity of sera from the eight subjects whose sequences were analyzed in this study. TRA= Transmission-reducing activity. TBA=Transmission blocking activity.

	K_D (x 10^{-10} ± SEM M)	k_a (x 10^{5} ± SEM 1/Ms)	k_{dis} (x 10^{-4} ± SEM 1/s)	N
**LMIV230-01**				
**Biological Replicate 1**	1.58 ± 0.77	1.71 ± 0.06	0.28 ± 0.15	3
**Biological Replicate 2**	2.06 ± 0.99	1.80 ± 0.04	0.37 ± 0.18	3
**LMIV230-02**				
**Biological Replicate 1**	6.36 ± 0.24	7.67 ± 0.21	4.87 ± 0.06	3
**Biological Replicate 2**	4.27 ± 0.22	6.37 ± 0.13	2.71 ± 0.10	3

Supplementary Table 2| Binding of mAbs LMIV230-01 and LMIV230-02 to Pfs230D1 using Biolayer Interferometry. Binding data for each mAb was fitted using a 1:1 binding model. The averages for two biological replicates, composed of three technical replicates each, are shown for both mAbs.

	Pfs230D1-LMIV230-01scFv
**Data collection**	
Space group	$P3_1$
Cell dimensions	
a, *b*, *c* (Å)	144.02 144.02 54.53
α, β, γ (°)	90.00 90.00 120.00
Resolution (Å)	19.74-2.00 (2.07-2.00)
R_{meas}	0.16 (0.80)
I/σ	5.34 (1.40)
CC(1/2)	98.70 (58.90)
Completeness (%)	99.43 (98.54)
Redundancy	3.22
**Refinement**	
Resolution (Å)	19.74 – 2.0
No. reflections	84,989
R_{work} / R_{free}	22.31/18.24
No. atoms	
Protein	9,435
Water	1,081
B-factors	
Protein	34.26
Water	39.66
R.m.s. deviations	
Bond lengths (Å)	0.014
Bond angles (°)	1.82
Validation	
MolProbity score	1.35
Clashscore	3.61
Poor rotamers (%)	0.00
Ramachandran plot	
Favored (%)	96.80
Allowed (%)	3.20
Disallowed (%)	0.00

Supplementary Table 3| Data Collection and Refinement Statistics

Residues in Pfs230D1	Interaction	Interface with LMIV230-01scFv	Residues in LMIV230-01scFv	CDRs
E565		Light	Q1	-
T566		Light	V2	-
T567		Heavy, Light	G27	H1
E568		Heavy	T28	H1
S569	H-bond	Heavy	N30	H1*
G570		Heavy	T31	H1*
D571		Light	Y32	H1
A573		Heavy, Light	A33	H1
V574		Heavy	I35	H1*
S575		Heavy	A50	H2
E576	H-bond & Salt bridge	Heavy	I52	H2
D577		Heavy	F54	H2*
Y579		Heavy	Q55	H2*
K581		Heavy	D56	H2*
K610		Heavy	R57	H2*
E612		Heavy	Q59	H2*
K653	H-bond	Heavy	K74	-
E655	H-bond	Heavy, Light	K98	H3*
T656		Heavy, Light	E99	H3

K657	H-bond	Heavy, Light	S100	H3
K698		Heavy	G101	H3
A699		Heavy	R102	H3
T700		Heavy	A103	H3
V701		Heavy	R107	H3*
Y703		Heavy	S173	L1
E724		Heavy	W175	L1
Y726		Heavy	Y192	L2
			K193	L2
			T196	L2
			L197	L2
			Y234	L3
			K235	L3*
			S236	L3
			Y237	L3
			W239	L3

Supplementary Table 4| Contact residues between Pfs230D1 and LMIV230-01scFv. Asterisks (*) refer to residues different from germline. CDRs= complementarity-determining regions.

Polymorphisms	Antibody contact	Frequency (MalariaGen)	Frequency (%)
G605S or G605R	-	*2,296 / 2,512*	*91.49 %*
K644Q	-	*1 / 2,512*	*0.04 %*
T652R	-	*50 / 2,512*	*1.99 %*
E654K	-	*1 / 2,512*	*0.04 %*
E655V	+	*6 / 2,512*	*0.24 %*
K661N	-	*550 / 2,512*	*21.89 %*
V687I	-	*1 / 2,512*	*0.04 %*
A699T	+	*5 / 2,512*	*0.20 %*
D714N	-	*1 / 2,512*	*0.04 %*

Supplementary Table 5| Polymorphisms within Pfs230D1. Table was generated using 2,512 sequences of *P. falciparum* available at MalariaGen.

		K_D (x 10^{-10} ± SEM M)	k_a (x 10^6 ± SEM 1/Ms)	k_{dis} (x 10^{-5} ± SEM 1/s)	N
**T652R**	Biological Replicates 1	1.50 ± 0.56	9.89 ± 0.9	0.96 ± 0.45	3
	Biological Replicates 2	1.40 ± 0.59	12.12 ± 0.36	1.7 ±0.73	3
**A699T**	Biological Replicates 1	0.01 ± 0.00	10.34± 0.41	0.01 ± 0.00	3
	Biological Replicates 2	0.07 ± 0.06	10.31 ± 1.03	0.07 ± 0.06	3
**E655V**	Biological Replicates 1	2.17 ± 0.33	9.15 ± 0.34	1.99 ± 0.33	3
	Biological Replicates 2	1.20 ± 0.20	13.47 ± 0.42	1.62 ± 0.26	3
**K661N**	Biological Replicates 1	0.16 ± 0.15	8.16 ± 0.47	0.12 ± 0.12	3
	Biological Replicates 2	1.10 ± 0.18	11.2 ± 0.45	0.72 ± 0.35	3
**E654K**	Biological Replicates 1	0.51 ± 0.07	9.81 ± 0.78	0.50 ± 0.10	3
	Biological Replicates 2	0.76 ± 0.03	11.40 ± 0.57	0.86 ± 0.04	3

Supplementary Table 6| Binding affinities of LMIV230-01 to Pfs230D1 variants as determined by BLI. Binding data were fitted using a 1:1 binding model. The averages for three technical replicates are shown.

LMIV230-01	LMIV230-02
**heavy chain variable region**	**Heavy chain variable region**
Q V Q L V Q S G A E V K K P G S S V K V S C K V S G G T F N T Y A I I W V R Q A P G Q G L E W M G A I I P F Q D R G Q Y A Q K F Q G R V T I T A D K S T S T A Y M E L S S L R S E D T A V Y Y C A K E S G R A V A D R W G Q G T L V T V S S	Q V Q L V Q S G A E V K K P G S S V K V S C K A S G G T F N S Y G I S W V R Q A P G Q G L E W V G G I I P S F G T A D Y A Q K F Q G R V T F T T D T S T S T A Y M E V S S L R S E D T A V Y Y C A G S S W S S G T Y Y G R S A Y W G Q G T L V T V S S
**Kappa chain variable region**	**Kappa chain variable region**
D I V M T Q S P A S L A L S L G E R A T L Y C R A S H S I S S W L A W Y Q Q K P G K A P K L L I Y K A S T L E S G V P S R F S G S G S G T E F T L T I S S L Q P D D F A T Y Y C Q Q Y K S Y P W T F G Q G T K L E I K R	E I V M T Q S P A S L A L S L G E R A T I N C K S S Q S V L Y S S N N K N Y L A W Y Q Q K P G Q P P K L L I Y W A S T R E S G V P D R F S G S G S G T D F T L T I S S L Q A G D V A V Y H C Q Q Y Y S T P Y S F G Q G T K L E I K R

Supplementary Table 7| Sequences of heavy and light chains correspondent to LMIV230-01 and LMIV230-02 mAbs.

MBFor1	5' GAG GTG AAG AAG CCT GGG TCC TCG GTG AAG GTC TCC TGC AAG GCT
MBRev1	5' GGT GCT AGC TGA GGA GAC CGT GAC CAG
MBFor2	5' CAC ACG AAT TCG CAG GTC CAG CTG GTG CAG TCT GGG GCT GAG GTG AAG AAG CCT GGG
MBfornodig1	5' AGT CTT GCA CTT GTC ACG AAT TCG CAG GTC CAG CTG GTG CAG TCT
MBrevnodig1	5' TGG GCC CTT GGT GCT AGC TGA GGA GAC GGT CAC CAG
MBkapfor1	5' TCC CTG GCT GTG TCT CTG GGC GAG AGG GCC ACC ATC AAC TGC A
MBkaprev1	5' CTC CGT ACG TCG TTT GAT CTC CAG CTT GGT
MBkaprev2	5' ACG AAT TCA GAC ATC GTG ATG ACC CAG TCT CCA GAC TCC CTG GCT GTG TCT C
MBkapfornodig1	5' AGT CTT GCA CTT GTC ACG AAT TCA GAM ATC GTG ATG ACC CAG TCT
MBkaprevnodig1	5' AGA TGG TGC AGC CAC CGT TCG TTT GAT CTC CAG CTT

Supplementary Table 8| Primers used to complete sequence of antibody framework regions.
