# Supplementary material for: A human monoclonal antibody blocks malaria transmission and defines a highly conserved neutralizing epitope on gametes
Source: Nat Commun. 2021 Mar 19;12:1750. doi: 10.1038/s41467-021-21955-1 (PMC7979743; doi:10.1038/s41467-021-21955-1)
Supplement: Supplementary file 2 — Reporting Summary [file 41467_2021_21955_MOESM2_ESM.pdf]

nature research

Corresponding author(s): Patrick E. Duffy

Last updated by author(s): 02/16/2021

Reporting Summary

Nature Research wishes to improve the reproducibility of the work that we publish. This form provides structure for consistency and transparency in reporting. For further information on Nature Research policies, see our Editorial Policies and the Editorial Policy Checklist.

Statistics

For all statistical analyses, confirm that the following items are present in the figure legend, table legend, main text, or Methods section.

n/a	Confirmed	
☐	☒	The exact sample size (*n*) for each experimental group/condition, given as a discrete number and unit of measurement
☐	☒	A statement on whether measurements were taken from distinct samples or whether the same sample was measured repeatedly
☐	☒	The statistical test(s) used AND whether they are one- or two-sided<br>*Only common tests should be described solely by name; describe more complex techniques in the Methods section.*
☐	☒	A description of all covariates tested
☐	☒	A description of any assumptions or corrections, such as tests of normality and adjustment for multiple comparisons
☐	☒	A full description of the statistical parameters including central tendency (e.g. means) or other basic estimates (e.g. regression coefficient) AND variation (e.g. standard deviation) or associated estimates of uncertainty (e.g. confidence intervals)
☐	☒	For null hypothesis testing, the test statistic (e.g. *F*, *t*, *r*) with confidence intervals, effect sizes, degrees of freedom and *P* value noted<br>*Give P values as exact values whenever suitable.*
☒	☐	For Bayesian analysis, information on the choice of priors and Markov chain Monte Carlo settings
☒	☐	For hierarchical and complex designs, identification of the appropriate level for tests and full reporting of outcomes
☐	☒	Estimates of effect sizes (e.g. Cohen's *d*, Pearson's *r*), indicating how they were calculated

Our web collection on statistics for biologists contains articles on many of the points above.

Software and code

Policy information about availability of computer code

Data collection	FlowJow v.9 was used to analyze the flow cytometry data for B cell populations.<br>-iRmap v.1 (Wang et al.) from iRepertoire was used to stitch and map sequencing reads from single cells. iPair files were analyzed using the -iPair Analyzer Gui v.2 (freely available for download) and exported to csv for further analysis in Microsoft Excel.
Data analysis	- IMGT/HighV-QUEST (2018) (online tool - used for sequence annotation)<br>- ChangeO 0.4.1 (open source tool - used for processing IMGT output)<br>- alakazam 0.2.8 (open source tool - used for drawing trees)<br>- msa 1.8.0 (open source tool - used for performing and visualising the sequence alignments)<br>- ggplot 2.2.1 (open source tool - used for making bar charts for clonotype genes)<br>- a custom script was used for clustering sequences - this can be made available on request<br>- GraphPrism7.0 was used for statistical analyses and graph construction<br>- XDS, Phenix 1.13, Refmac 7.0.076, Coot 0.8.9.2 and Pymol 1.2 were used for X-ray structure data processing, structure refinement and structure visualization

For manuscripts utilizing custom algorithms or software that are central to the research but not yet described in published literature, software must be made available to editors and reviewers. We strongly encourage code deposition in a community repository (e.g. GitHub). See the Nature Research guidelines for submitting code & software for further information.

Data

Policy information about availability of data

All manuscripts must include a data availability statement. This statement should provide the following information, where applicable:

- Accession codes, unique identifiers, or web links for publicly available datasets
- A list of figures that have associated raw data
- A description of any restrictions on data availability

All data generated or analyzed during this study are included in this published article (and its Supplementary Information files). Raw sequencing data are available on request from the corresponding author. Atomic coordinates and structural factors of Pfs230D1-LMIV01-scFv were deposited to the Protein Data Bank under accession code 7JUM.

Field-specific reporting

Please select the one below that is the best fit for your research. If you are not sure, read the appropriate sections before making your selection.

- [x] Life sciences
- [ ] Behavioural & social sciences
- [ ] Ecological, evolutionary & environmental sciences

For a reference copy of the document with all sections, see nature.com/documents/nr-reporting-summary-flat.pdf

Life sciences study design

All studies must disclose on these points even when the disclosure is negative.

Sample size	No sample size calculation was performed, as we chose subjects from the vaccine trial presenting higher antibody titers and functional activity for production of monoclonal antibodies.
Data exclusions	No data were excluded from this study.
Replication	Experiments were performed at least in duplicate. All attempts at replication were successful.
Randomization	Randomization is not relevant in this study as for mAb expression we chose the subjects presenting high antibody titers and functional activity in response to Pfs230D1-EPA/Alhydrogel.
Blinding	The ELISA and SMFA assays performed on the serum samples were conducted in a blinded manner. Blinding of the investigators was not applied for other aspects of the study that examined functional and structural aspects of individual human mAbs.

Reporting for specific materials, systems and methods

We require information from authors about some types of materials, experimental systems and methods used in many studies. Here, indicate whether each material, system or method listed is relevant to your study. If you are not sure if a list item applies to your research, read the appropriate section before selecting a response.

Materials & experimental systems

n/a	Involved in the study	
[ ]	[x]	Antibodies
[ ]	[x]	Eukaryotic cell lines
[x]	[ ]	Palaeontology and archaeology
[x]	[ ]	Animals and other organisms
[x]	[ ]	Human research participants
[ ]	[x]	Clinical data
[ ]	[ ]	Dual use research of concern

Methods

n/a	Involved in the study	
[x]	[ ]	ChIP-seq
[ ]	[x]	Flow cytometry
[x]	[ ]	MRI-based neuroimaging

Antibodies

Antibodies used	For Flow Cytometry, human PBMCs were stained with the following surface-conjugated antibodies: CD3 (UCHT1), CD14 (M5E2), CD56 (HCD56) Alexa<br>Fluor 700, CD19 APC-CY7 (HIB19) CD20 PE-CY7 (2H7) and CD27 APC (LG.3A10) purchased from Biolegend (San Diego, USA). \<br>LMIV230-01 and LMIV230-02 mABs were expressed in HEK293 cells. For confocal imaging, the mAb anti-C5b-9 + C5b-8 was purchased from Abcam, (ref ab66768) and used at 10ug/mL .
Validation	*Describe the validation of each primary antibody for the species and application, noting any validation statements on the manufacturer's website, relevant citations, antibody profiles in online databases, or data provided in the manuscript.*

April 2020

Eukaryotic cell lines

Policy information about cell lines

Cell line source(s)	Expi293 cells were obtained from Thermo Fisher and were used for expression of antibodies.
Authentication	All of the cell lines used were obtained commercially with a certificate of analysis
Mycoplasma contamination	The cell lines were not tested for mycoplasma contamination
Commonly misidentified lines (See ICLAC register)	No commonly misidentified cell lines were used.

Clinical data

Policy information about clinical studies

All manuscripts should comply with the ICMJE guidelines for publication of clinical research and a completed CONSORT checklist must be included with all submissions.

Clinical trial registration	NCT02334462
Study protocol	The protocol will be made publicly available when the final Clinical Study Report is submitted to the FDA.
Data collection	The study was conducted by the Malaria Research and Training Center (MRTC), University of Bamako at the John Lamontagne Malaria Research Center in Bancoumana, a village in Mali. The study was conducted from April 2015 to October 2017.
Outcomes	Safety outcomes will be local and systemic adverse events (AEs) and serious adverse events (SAEs). Immunogenicity outcomes will be antibody responses as measured by ELISA against recombinant Pfs25, Pfs230, and EPA, and B cell responses. Functional activity of the induced antibodies will be assessed by membrane feeding assays conducted at the National Institute of Allergy and Infectious Diseases in the US.

Dual use research of concern

Policy information about dual use research of concern

Hazards

Could the accidental, deliberate or reckless misuse of agents or technologies generated in the work, or the application of information presented in the manuscript, pose a threat to:

No	Yes	
☒	☐	Public health
☒	☐	National security
☒	☐	Crops and/or livestock
☒	☐	Ecosystems
☒	☐	Any other significant area

Experiments of concern

Does the work involve any of these experiments of concern:

No	Yes	
☒	☐	Demonstrate how to render a vaccine ineffective
☒	☐	Confer resistance to therapeutically useful antibiotics or antiviral agents
☒	☐	Enhance the virulence of a pathogen or render a nonpathogen virulent
☒	☐	Increase transmissibility of a pathogen
☒	☐	Alter the host range of a pathogen
☒	☐	Enable evasion of diagnostic/detection modalities
☒	☐	Enable the weaponization of a biological agent or toxin
☒	☐	Any other potentially harmful combination of experiments and agents

Flow Cytometry

Plots

Confirm that:

☒ The axis labels state the marker and fluorochrome used (e.g. CD4-FITC).

☒ The axis scales are clearly visible. Include numbers along axes only for bottom left plot of group (a 'group' is an analysis of identical markers).

☒ All plots are contour plots with outliers or pseudocolor plots.

☒ A numerical value for number of cells or percentage (with statistics) is provided.

Methodology

Sample preparation	PBMCs were thawed in 37°C water bath, resuspended in complete RPMI and washed with PBS. 1 µM of BSA-decoy tetramer was added to 2 to 3 million PBMCs and incubated at room temperature for 5 minutes, protected from light. Pfs230D1M-PE tetra mer at lµM was added to the cells that were then incubated at 4QC for 20 minutes. Cells were washed with PBS containing 10% FBS and incubated with 25 µL of anti-PE magnetic beads (Miltenyi Biotech, Bergisch Gladbach, Germany) for 25 minutes. Four ml of PBS were added to the solution, which was then passed over magnetized LS columns for elution of cell suspension enriched for Pfs230D1M-PE cells. After enrichment with Pfs230D1M tetra mer, human cells were stained with the following surface-conjugated antibodies: CD3 (UCHTl), CD14 (M5E2), CD56 (HCD56) Alexa Fluor 700, CD19 APC-CY7 (HIB19) CD20 PE-CY7 (2H7) and CD27 APC (LG.3A10) purchased from Biolegend (San Diego, USA).
Instrument	Analysis was performed in FACSAria'" II instrument (Fluorescence-activated cell sorting, BO Biosciences, San Jose, USA)
Software	BO FACSDivaTM software
Cell population abundance	Cell population abundance is expressed at Supplementary Figure 2.
Gating strategy	A gating strategy was performed first for doublet discrimination, then singlet cells were selected for exclusion of non-B cells using CD3, CD14 and CD56 markers. Lymphocytes were gated for CD19+c020+. Further discrimination with CD27+ allowed gating for memory B cells. Pfs230D1M-specific B cells were gated using PE and excluding non-Pfs230D1M cells gated using CF594, the fluorochrome used in the decoy BSA tetra mer.

☒ Tick this box to confirm that a figure exemplifying the gating strategy is provided in the Supplementary Information.
